# Learning in Transcriptional Network Models: Computational Discovery of Pathway-Level Memory and Effective Interventions

**DOI:** 10.3390/ijms24010285

**Published:** 2022-12-23

**Authors:** Surama Biswas, Wesley Clawson, Michael Levin

**Affiliations:** 1 Allen Discovery Center, Tufts University, Medford, MA 02155, USA; 2 Department of Computer Science & Engineering and Information Technology, Meghnad Saha Institute of Technology, Kolkata 700150, India; 3 Wyss Institute for Biologically Inspired Engineering, Harvard University, Boston, MA 02115, USA

**Keywords:** biological network, training, memory, association, pharmacoresistance, sensitization

## Abstract

Trainability, in any substrate, refers to the ability to change future behavior based on past experiences. An understanding of such capacity within biological cells and tissues would enable a particularly powerful set of methods for prediction and control of their behavior through specific patterns of stimuli. This top-down mode of control (as an alternative to bottom-up modification of hardware) has been extensively exploited by computer science and the behavioral sciences; in biology however, it is usually reserved for organism-level behavior in animals with brains, such as training animals towards a desired response. Exciting work in the field of basal cognition has begun to reveal degrees and forms of unconventional memory in non-neural tissues and even in subcellular biochemical dynamics. Here, we characterize biological gene regulatory circuit models and protein pathways and find them capable of several different kinds of memory. We extend prior results on learning in binary transcriptional networks to continuous models and identify specific interventions (regimes of stimulation, as opposed to network rewiring) that abolish undesirable network behavior such as drug pharmacoresistance and drug sensitization. We also explore the stability of created memories by assessing their long-term behavior and find that most memories do not decay over long time periods. Additionally, we find that the memory properties are quite robust to noise; surprisingly, in many cases noise actually increases memory potential. We examine various network properties associated with these behaviors and find that no one network property is indicative of memory. Random networks do not show similar memory behavior as models of biological processes, indicating that generic network dynamics are not solely responsible for trainability. Rational control of dynamic pathway function using stimuli derived from computational models opens the door to empirical studies of proto-cognitive capacities in unconventional embodiments and suggests numerous possible applications in biomedicine, where behavior shaping of pathway responses stand as a potential alternative to gene therapy.

## 1. Introduction

Brains enable remarkable behavioral capacities and are required for the complex cognitive abilities and first-person experience that evolved human and other animals possess. However, it is critical to remember that each of us has taken the journey across the Cartesian Cut [1]—smoothly and slowly transforming from a bag of biochemical reactions (a quiescent oocyte) into an adult form, capable of rational thought, metacognition, and a sense of selfhood as distinct from “insentient objects”. To the extent that “ontogeny recapitulates phylogeny” [2], taking evolution and developmental biology seriously means seeking to understand primitive forms of cognition as a spectrum that could extend to unconventional substrates besides mature complex brains. Recent progress in the field of basal cognition seeks phylogenetic antecedents to memory and other cognitive functions and asks what minimal dynamics are sufficient for implementing simple functional building blocks that underlie more advanced minds [3,4,5,6,7]. A key aspect of this research axis, related to not only evolutionary biology but also synthetic bioengineering, exobiology, and robotics/AI, is the development of frameworks for recognizing proto-cognitive activity in unfamiliar embodiments [8,9].

Morgan’s Canon [10] urges us to err on the side of mechanistic, over cognitive, models of biological systems. However, in the case of systems that are capable of learning, having preferences, or even more advanced functions, treating them as simple machines that can only be modified at the hardware level is often a sub-optimal strategy. Humans have discovered, by training animals for millennia (despite knowing no neuroscience), that effective prediction and control in some systems can be achieved by taking advantage of the system’s competencies, such as learning. This allows manipulation through an interface different than hardware rewiring: that of stimuli. Recently, we proposed an explicit framework for the empirical investigation of systems (of evolutionary or engineered origin) based on what level of cognitive model avails the engineer the most efficient rational control over the system’s function [11]. We proposed that the same efficiency and causal power gains that result from training animals in such a way, rather than micromanaging their neural states, can be reaped in biomedicine [12,13,14]. Moreover, we suggested that behavior takes place not only in 3D space by canonical muscle motion, but also in metabolic, transcriptional, and anatomical spaces [15]. Thus, it may be possible to achieve high levels of control in manipulating health and disease by behavior-shaping the dynamic navigation of living systems within any of these spaces. These strategies offer a key to the inverse problem (i.e., what low-level rules to alter to achieve a system-level change [16]) plaguing genome-editing and molecular medicine. They may also enable the development of strategies that take synthetic biology beyond simple circuits and into complex bioengineering [17,18,19,20,21]. How many of the tools of behavior science [22] can be brought to bear on cells, tissues, and other body components that are not brains, is an empirical question.

Protein pathways and gene regulatory networks (GRNs), as well as many other chemical pathways, are key drivers of embryogenesis [23], cell behavior, and complex physiology [24,25]. Understanding the dynamics of these pathways is important not only for the study of evolutionary developmental biology [26,27,28,29,30], but also for the prediction and management of numerous disease states including cancer [31,32,33]. Thus, effort has gone into computational inference of both protein pathways and GRN models [34,35,36], and the development of algorithms for predicting their behavior [37]. In general, these pathways are conventionally treated as a simple machine with most strategies focusing around rewiring their structure to achieve a desired outcome. While dynamical systems approaches have made great strides in understanding how these systems settle on specific stable states [38,39], the prospects for designing interventions have lagged: it is often unclear how to edit the structure of the pathway to result in the appropriate dynamic behavior (or in the case of developmental GRNs, the desired anatomy). Indeed, some approaches have aimed at control of these networks at the structural level [40], others, using Boolean network models, have shown control measures based on perturbations of specific nodes identified through network measures [41,42]. While this prior work examined changes to the *physical structure* of networks as learning, here we specifically focus on dynamical systems learning that requires no alteration to the properties of the network components [43]—a desirable property from the therapeutic perspective. Likewise, there exist formalisms that address the control of certain biological networks towards larger scale phenotypic goals, such as cell fates [44,45,46,47]. However, frameworks are still needed that facilitate the discovery of interventions for altering system-level dynamics (such as those provided efficiently by training regimens in behavioral science).

An alternative hypothesis concerns the utility of applying a computational lens to GRNs and other biological networks [48], seeing them as agents which convert activation levels of certain genes (inputs) to those of effector genes (outputs), with layers of other nodes between them. A view of pathways as an information-processing device of unknown computational capacity, as opposed to a mechanical clockwork, would therefore suggest strategies to control network behavior via inputs. Under this view, spatiotemporally regulated patterns of stimuli could remodel the landscape of attractors corresponding to a system’s “memory”. Many systems, from molecular networks [43,49] to physiological networks in somatic organs [50,51] exhibit plasticity and history-based remodeling of stable dynamical states. Consistent with the basal cognition paradigm, we define memory in functionalist terms—a system in which future behavior is specifically modulated by past experience. This definition holds regardless of the system’s physical implementation and is not limited to canonical views of behavioral memory, such as changes at the epigenetic and protein levels [52,53,54,55], or the requirement of synapses and neurons. It is an engineering perspective, in which memory in diverse media is a broader phenomenon and is not restricted to its common manifestations. Such a substrate-agnostic view enables the question: could trainability be found in molecular networks?

**Figure 1:**
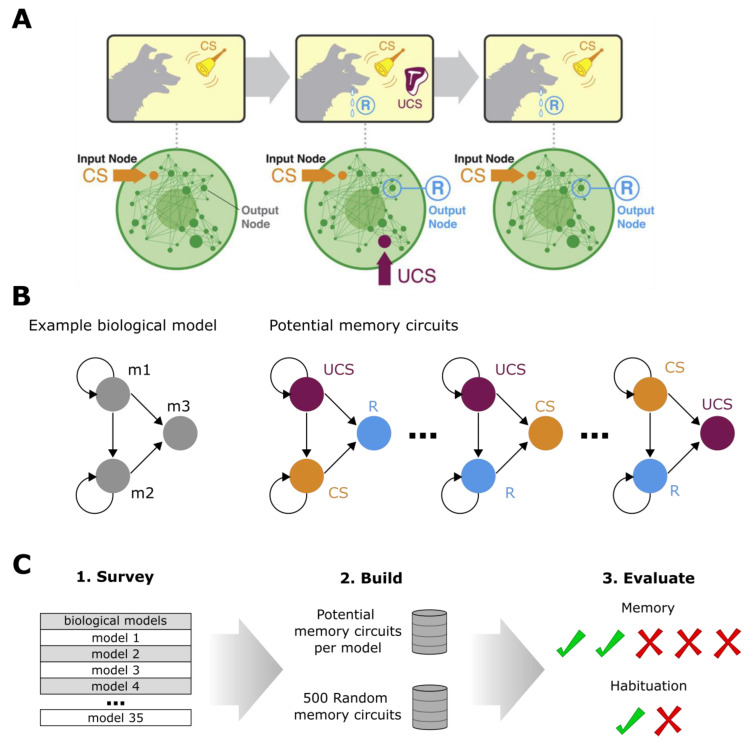
**Conceptual approach and experimental design.** (**A**) A cartoon representation of applying concepts from behavioral science, such as classical conditioning, towards controlling a biological network. Knowledge is not needed of how a dog’s brain changes during the training session that pairs the sound of a bell (stimulus) to a drooling response, but it is possible to form such a memory. In the same way, we need not understand the intricacies of a biological network in order to train a stimulus to a response. (**B**) Given a biological network model, such as the example on the far right, potential memory circuits are built that test the model with every possible pairing of stimulus (UCS, violet), response (R, blue), and potential conditioned stimulus (CS, yellow). The number of potential memory circuits is therefore the number permutations of N nodes with three choices (i.e., six in this cartoon). (**C**) Cartoon of analytical pipeline. Thirty-five biological models are used to build both potential memory circuits as well as 500 random networks. All networks are tested for memory, habituation, and sensitization (not shown). (**A**) is adapted from [67], with permission.

Several prior studies have tested specific memory phenomena in GRN models [56,57,58,59,60,61,62,63,64,65]. Others have addressed harnessing dynamical systems approaches to control networks through the structure of the network interactions such as feedback loops [66]. However, it is still largely unclear what kind of memories various pathways can possess, and whether it is possible to use this knowledge to drive the discovery of interventions. In past work [67], we formalized the notion of training a chemical pathway via pulsed input stimuli (node activation or suppression drugs), and defined a taxonomy of memory types appropriate to the GRN system. We demonstrated how paradigms from behavior science can be naturally applied to cell-biological contexts (Figure 1A). Specifically, we developed an algorithm that does not rewire an existing network but interrogates the natural system to discover the optimal perspective from which it can be controlled with existing ‘training tools’, such as Pavlovian conditioning. This, for example, was achieved by computationally identifying which transcriptional loci can serve as efficient neutral stimulus (NS) and unconditioned stimulus (UCS) nodes in the GRN network, such that the desired response (R) can be seen in a chosen gene’s expression level after training regimes such as Pavlovian conditioning. We therefore can investigate any network of size N by testing every permutation of NS, UCS, and R (Figure 1B) and evaluating memory of each possible permutation (Figure 1C).

Here, we sought the following extensions of prior work. First, we explore continuous (ODE) models to show that the phenomena we found were not due to a Boolean nature of previously explored models. Second, we analyze and compare continuous models of not only transcriptional networks, but both protein signaling pathways and GRNs, with randomized models to show that evolved systems possess different learning properties than those that can be obtained by chance using similar network properties [68,69]. Third, we test the long-term stability of memories by assessing their behavior over long time scales, to determine whether memory declines over time. Fourth, we characterize their robustness to stochastic noise, and in general to different strengths of input stimuli. Finally, having found examples of behavior such as habituation (decreased response to repeated stimulation) and sensitization (increased response to repeated stimulation), graded properties that are not detectable in binary or Boolean networks, we demonstrate the existence of specific stimuli regimes that abolish these memories. These results provide a possible strategy to combat pharmacoresistance and other prevalent problems limiting use of pharmacological agents in biomedicine [70,71,72,73]. Importantly however, the memory properties of these networks are not meant to be only a road toward therapeutics—they are expected to be an important part of their function in normal scenarios, making them interesting to both evolutionary and developmental biology. Overall, we show that a computational perspective on a minimal model of memory and learning can contribute toward a roadmap for the development of biomedical interventions that do not require genomic editing, gene therapy, nor any structural modification of the network itself.

## 2. Results

### 2.1. Biological Networks May Possess Multiple Types of Memory

Biological pathways represent mutual interactions among molecular entities such as genes, proteins, and metabolites within a given cellular context and link to potentially many different biological processes. Here, we survey 35 published biological pathway networks from BioModels (see Table S1) [74,75,76,77,78,79,80,81,82,83,84,85,86,87,88,89,90,91,92,93,94,95,96], a mathematical model repository [97,98,99,100,101,102,103,104]. Each model is comprised of genes, proteins, or metabolites which are represented as nodes and the mutual chemical reactions are defined by edges. The dynamics of the chemical reactions themselves are comprised within an ordinary differential equation (ODE) that gives the rate of change of the expression level of a node over time. Depending on the given initial expression level and current time instance, the expression level of a node is evaluated by solving these ODEs. To model an external input to a given node, we can mathematically alter the expression of the biomolecule, allowing its use as a stimulus (conceptually similar to applying sensory input to an animal for training), applying either stimulation (increased expression level) or inhibition (decreased expression level). Then, considering a separate node’s expression level as a sensory response, we analyze whether it was up- or downregulated by the applied stimulus. We built a memory ‘test suite’ to run across all biological pathway models to determine whether, and which, memory phenotypes (transfer memory, consolidation memory, etc.) are found [65]. In short, for each memory model three nodes are selected to be an unconditional stimulus (UCS), a potentially conditioned stimulus (CS), and a response (R). For each of the 35 models, a set of potential memory models is built out of all possible combinations of the n-node model. For example, a three-node network has six potential memory models. We then tested each potential memory network for each surveyed biological pathway, evaluating the different combinations of nodes in different roles and recorded the types of memories found in the dynamics [67] (Figure 1, see Section 4 for more details).

**Figure 2:**
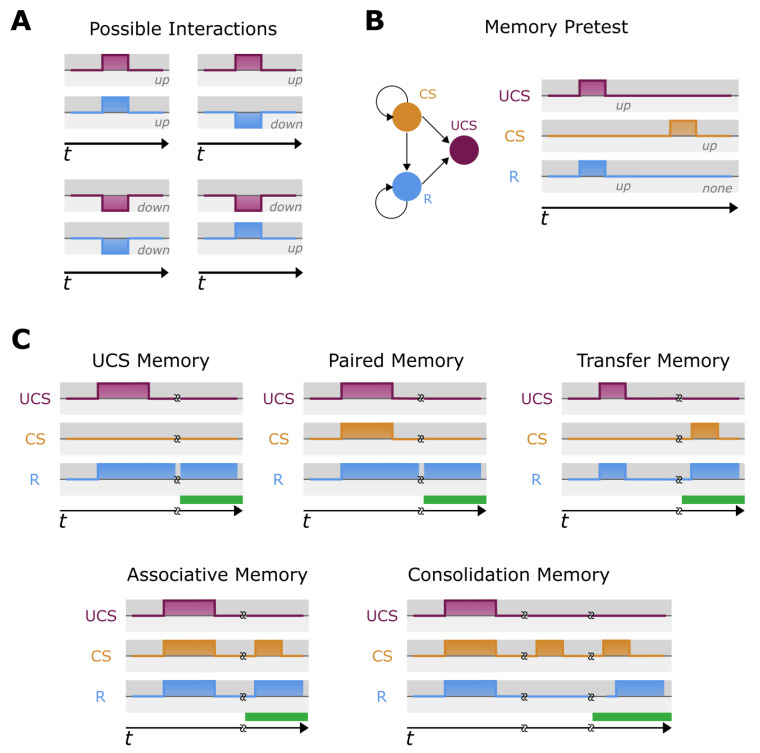
**Proposed memory types in biological networks.** (**A**) Graphical representation of possible scenarios between UCS and R; an upregulation of UCS leads to upregulation of R, an upregulation of UCS leads to downregulation of R, a downregulation of UCS leads to downregulation of R, or downregulation of UCS leads to upregulation of R. Note that stimulation may be either upregulation or downregulation. Likewise, a response may be upregulation or downregulation. Note that while all responses have the same intensity, this is for visualization only. (**B**) The memory pretest for each potential memory circuit. First, UCS is stimulated, and the R node checked for response. Second, the chosen CS node is stimulated, and the R node is checked to ensure no response occurs. If any of these tests fail, the potential memory circuit is rejected. (**C**) The five tested memory phenotypes for all potential memory tests that pass the pretest (see description in Section 4). While responses are shown to be simultaneous this is not the case in normal biomodels, and we note that in this way for visual clarity only. Colors are matched to node identity and colors in the area under the stimulation or response are for visualization. The green bar represents the ‘test period’, where the desired memory responses is searched for after a stimulation of CS.

We define the existence of memory in an ODE modeled biological network as altered, persistent future expression of a given biomolecule in response to the stimulation of another biomolecule in the network. Our definition of memory in ODE-modeled biological networks resembles memory defined in the study of Boolean-modeled GRNs with a few differences [67] (see Section 3). Most crucially, binary network investigation considers ‘states’ of the node as binary variables, either off or on. While ODE models have theoretically infinite states, as they are continuous equations, we define them as having three potential modes: upregulated, downregulated, and non-regulated. Importantly, these modes are not discrete (i.e., −1, 0, or 1), but can have various degrees of regulation. Overall, this allows for more robust characterization and leads to four key relationships between nodes that are associated with our definition of memory: upregulation of a stimulus (ST) can upregulate a response (R), upregulation of ST can downregulate R, downregulation of ST can upregulate R, and downregulation of ST can downregulate R (Figure 2). We consider two types of stimuli: (1) unconditioned stimulus (UCS), which unconditionally regulates R, and (2) a neutral stimulus, which does not regulate R but can be converted to a conditioned stimulus (CS) upon proper training. It is important to note that we refer to this third stimulus always as CS for clarity, as a neutral stimulus has no effect on memory.

We evaluate five kinds of ODE network memory: UCS-based memory [65], pairing memory (PM), transfer memory [74], associative memory (AM), and consolidation memory (CM) (see Figure 2 for a visual description and Methods for explicit description). A UCS memory is when a strong training stimulus is delivered, causing a long-term stable response of R. While this is the simplest memory we evaluated, its usefulness from a bioengineering standpoint may be invaluable as a simple latch. A paired memory is similar to the UCS memory, but in this case the UCS and CS are stimulated simultaneously to see if a long-term stable response is caused in R. This is different from UCS memory, as this memory only needs one node to be stimulated, whereas a paired memory requires both. Paired memory can be viewed as a ‘fail-safe’ system or AND gate; a response is only generated when two nodes are stimulated, and therefore R is partially protected from noise (i.e., if one node is noisily regulated). Transfer memory occurs when the original UCS stimulation drives the network dynamics into a slightly different configuration, allowing a previously non-effecting node to now effect the response node. An associative memory is similar to the classical Pavlovian conditioning model wherein a previously non-effective stimulus is delivered alongside a stimulus which causes a response in R. One striking possibility for such a memory phenotype would be to associate a desired response from a potentially dangerous stimulus (as in a drug with a hazardous side effect) with a more accessible or less hazardous stimulus that previously did not affect the circuit. Finally, consolidation memory is the same paradigm as in associative memory, but there is a consolidation period before the CS creates a response on R. All of these types of memories have implications for how cells bearing such GRNs would behave in healthy physiology and disease states (in which case they can be exploited by therapeutic strategies). These provide a context-sensitive response to internal and external (environmental) stimuli that are sure to have impacts on evolution and systemic function.

Overall, we found memory models in all surveyed biological networks except four models, of which three had a low number of nodes (<4) suggesting that too small networks either cannot, or are very unlikely to, have memory (Networks 3, 14, 17 and 33, and Table S2). Additionally, we tested to see if certain network properties, such as mean number of nodes, edges, degree, and hubness were associated with more or less memory and found there were no significant correlations with any of these measures and any memory (see Discussion).

**Figure 3:**
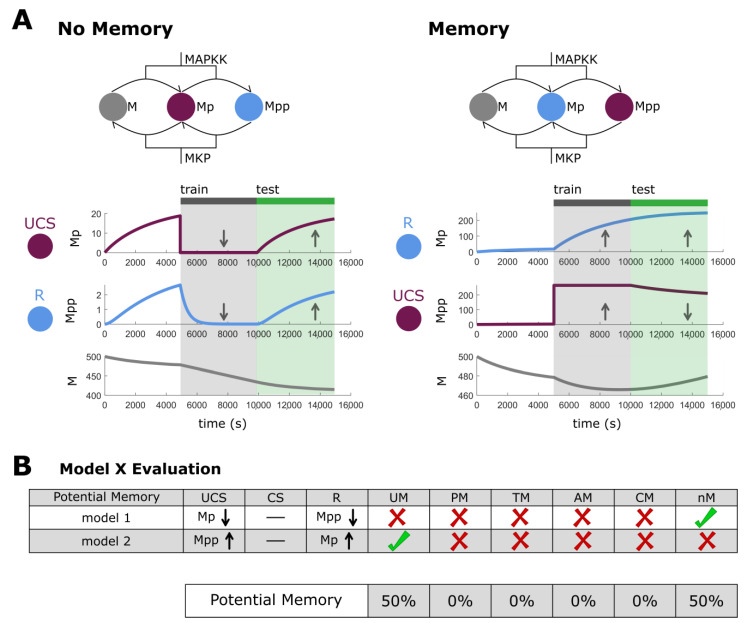
**Example network and memory evaluation.** (**A**) Two potential memory models derived from the sample biological network [75]. On the left, a UCS and R pairing that show no memory. The UCS (Mp) is stimulated (downregulated) during the grey training period and R (Mpp) is similarly downregulated. However, when the stimulation stops R reverts to its original behavior in the test period (green). On the right, the same network, but a different permutation of the UCS and R pairing which demonstrates memory. The UCS (Mpp) is stimulated (upregulated) and R (Mp) shows a response from its baseline behavior. However, when stimulation is halted (green test period), the behavior of R has continued to rise, rather than returning to baseline. Therefore, we conclude that this circuit shows a form of memory consistent with our functional definition—the behavior of R has been specifically modulated by past experience via stimulation of the UCS. (**B**) A cartoon representation of how potential memory models are evaluated using the example from (**A**). In this biological model, only two potential memory models passed the pretest and of those only one showed any memory (50%).

Before testing for memory, a pretest is performed to evaluate if the chosen UCS node can indeed cause a response on the R node while the chosen CS node elicits no response on the R node (Figure 2). The remaining circuits are then tested for the five kinds of memory as well as no memory. Each kind of memory is associated with a specific paradigm of training, i.e., sequences of stimulation which results in stable, long-lasting, predictable, and specific changes in network behavior that map nicely onto concepts of training in behavioral science. Stimulation can be used to train these networks, i.e., to shape their future behavior, just as is achieved in traditional biological systems. Visual examples of the five memories, their training, and tests can be seen in Figure 2 and details found within Methods. A ‘no memory’ circuit is defined as a circuit that has a UCS-R relationship, but no memory component (i.e., when UCS stimulation is removed, R reverts its expression to pre-stimulus expression levels). We show the evaluation process with an example three-node biological network [75] with two out of six potential memory circuits passing the pretest. We demonstrate that this model has UCS-based memory in one case and no memory in the other (Figure 3). To quantify this, the number of circuits showing a particular memory type are summed and divided by the number of potential memory circuits, giving the lower bound of 0% (no circuits with a given type of memory) and an upper bound of 100% (every circuit demonstrating that type of memory). Therefore, the example network shows a 50% memory capacity (one out of two networks) and a 50% no memory capacity (one out of two networks).

**Figure 4:**
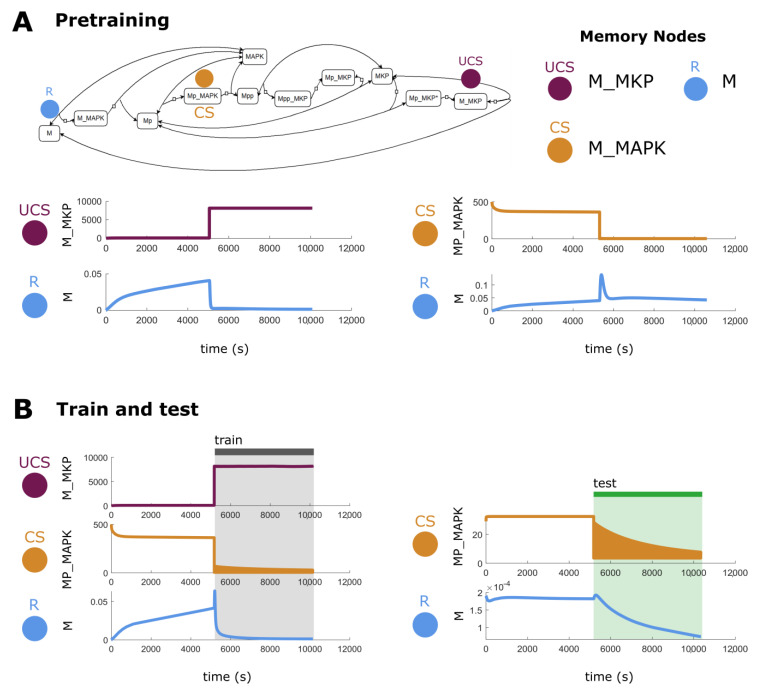
**Associative memory in a biological network.** (**A**) An example of associative memory in a potential memory model derived from a biological model from [75]. This particular memory model passes the pretest—UCS stimulation leads to change in R while a stimulation of CS does not. (**B**) To train the model towards a response in R from a stimulation in CS, the UCS and CS are stimulated at the same time, upregulated and downregulated, respectively. After, the circuit is tested and now stimulation of the CS (downregulation) causes lasting change in R. The colors under the yellow line in both training and testing periods are meaningless and are artifacts due to the integrator used.

The idea of self-sustaining feedback loops in biochemical reaction networks and their relationship with hysteresis may be one explanation for simple memories [105]. Figure 3 shows the dual phosphorylation-dephosphorylation cycle of MAPK, which has been characterized as having positive feedback loop architecture [75]. This does not diminish, however, the ideas that memories have been formed through stimulation of key nodes in this feedback loop. For example, while simple, a traditional latch circuit, common in electrical engineering, works in a similar fashion but still can be thought of as a memory. Overall, we suggest that feedback loop network architecture may be conducive to memories but may be only one way of many to support them. In a different biological network [75], we show an example of AM, wherein a previously neutral stimulus is converted to a CS through training (Figure 4). Again, while feedback loop architecture is present, our results indicate that memories can be formed through dynamical interactions alone, without changing network structure/architecture. Additional examples of other memory types are presented in Figures S4–S7.

### 2.2. Memory Effects Are Robust to Different Strengths of Stimulus

We also sought to examine the effects of varying stimulation strengths during the training period, to determine whether therapeutic triggers would have to be used in a very narrow range of concentrations. We examined the persistence of memories given smaller stimulations, namely 10X and 50X of baseline, whereas normal training was completed with 100X (see S8A). While most models (52%) kept the same number of memories, 10% of models lost memories, with only one losing more than 50% of the memories found at 100X. Interestingly, four models demonstrated a 50% increase in memory with weaker simulation, which suggests that some models are more sensitive to strong stimulations (i.e., too strong and no memory is formed). We conclude that the memory properties are robust to a wide range of stimulus strengths, a desirable property for therapeutics, and also for functional linkages with other cell pathways during evolution.

### 2.3. Memory Effects Are Robust to Noise

To determine the sensitivity of the results to noise, which represents a more realistic view of how biology functions [106], we re-ran the analyses while adding stochasticity throughout the simulation (see S8B). Interestingly, while almost all models retained the memories created (83%), there was an overall 20% increase in memory in the presence of noise (averaged across models and memory phenotypes) most notably in the associative memory paradigm. We conclude that biological noise should not inhibit the trainability of most networks, and indeed actually seems to be helpful, a phenomenon familiar in many biological systems, including signaling pathways [107,108].

### 2.4. Memory Effects Are Stable over Long Periods

We examined the stability of memories of long periods of time, relative to the model, after training to determine how quickly memories might decay after experience. We investigate the long-term presence of UCS memory in two ways: (1) by testing R nodes after 10X the initial waiting period and then again after 100X times the initial waiting period, and (2) by testing the R nodes after 100X times the initial waiting period. This paradigm investigates both the stability of memories (2), as well as whether testing can potentially destroy the formed memory (1). Out of 30 networks showing UCS memory, a near majority did not lose significant memory capacity (defined as <50% loss) with any delay. Twelve networks did lose more than 50% of memories over long timeframes, including 1, 21, and 25, each of which experienced loss of 73%, 60%, and 53%, respectively. Of those losses, most occurred between 1 and 10X timepoints but were constant after that; only eight networks lost all memories across both 10X and 100X tests (S9). Interestingly, in two cases the memories increased after testing at 10X, suggesting that memory recall could potentially strengthen certain dynamics, but we emphasize that this effect was rare. Overall, we conclude that while some networks have a limit on how long they retain training, the majority of networks can retain the bulk of their possible memories over very long time periods and that in general, memory ‘access’ does not destroy the memory but can, in some rare cases, increase memory.

### 2.5. Can Networks Be Trained to Keep More Than One Memory?

The memory capacity of neural networks, such as Hopfield networks [109], have been thoroughly examined [110] and reveal that even small networks could have capacities larger than one. However, in prior work it was not known whether GRNs or protein pathways could be trained in a way that did not wipe out the effects of prior experience. Could a single system, or network, keep more than one memory?

**Figure 5:**
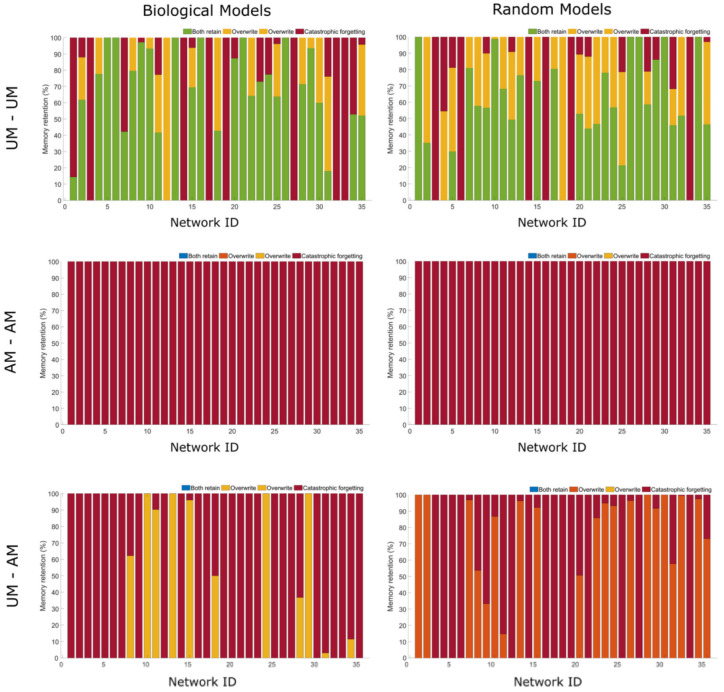
Serial Memories in Biological and Random Models.

**Figure 6:**
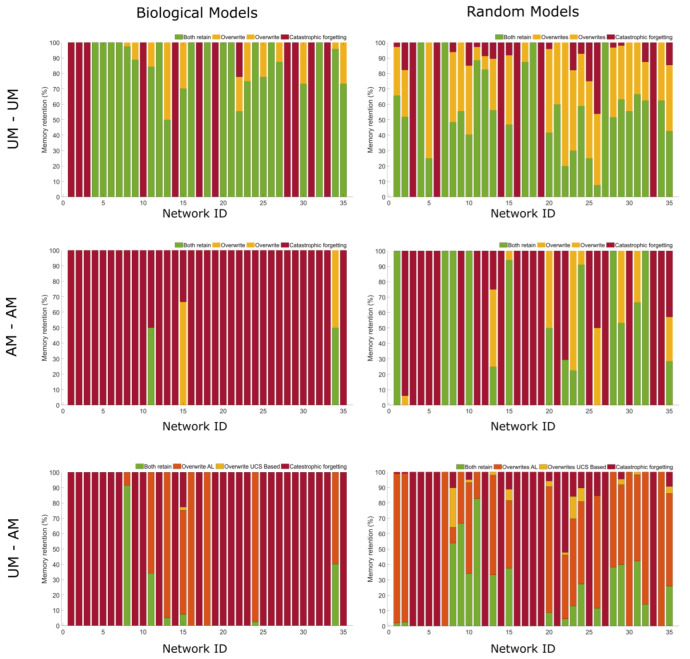
Parallel Memories in Biological and Random Models.

Thus, we tested all networks to determine whether a second memory could be added in addition to the first. Two paradigms of adding a secondary memory were used: (1) memories were added serially by giving training stimulations one after another allowing a short relaxation period and then giving a second training stimulation, and (2) memories were added in parallel by giving two training stimulations simultaneously and then tested. It is important to note that each memory requires a separate UCS-CS-R permutation, and therefore the functional roles of some nodes may be different between the two memories, may be the same, or may be partially overlapping. It however is never the case that the response node of one permutation is the UCS or CS of another. Interestingly, we find that both biological and random networks were able to add an additional UCS to a UCS memory in both cases of serial or parallel memories (Figure 5 and Figure 6). This may be due to the ‘simple’ nature of UCS memory, a stimulus creating a longer-term response. When more complex memories were tested, here AM-AM memory pairs, when created serially all biological and random models showed catastrophic forgetting wherein both are lost. When created in parallel, two biological networks retained both memories in some of their potential memory models while roughly half of random networks retained theirs. The case for UCS-AM memory pairs was similar, in that when created serially, both biological and random networks either forgot one or both, and when created in parallel random networks showed a stronger remembering of both memories (see Section 4). While it may be surprising that random networks are able to better form paired memories, especially in parallel, it may be that biological models have emerged to use certain dynamics towards a typical biological function and therefore may not have the capacity for additional memories such as we propose.

Both biological and random networks were tested for the capability of forming two memories, serially, or one after the other after a brief period of relaxation. We tested UM-UM memories, AM-AM memories, and UM-AM across all models. Above are bar graphs representing the percentage of potential memory models that either retain both memories, forget the first, or forget both. To test this, we followed a similar procedure as in memory testing. However, in this case once a memory was formed, another was formed following the same procedure (see Section 4). Note there was no pretest for the second memory, as this potential memory circuit had already passed.

Both biological and random networks were tested for the capability of forming two memories in parallel, or at the same time. We tested UM–UM memories, AM–AM memories, and UM–AM across all models. Above are bar graphs representing the percentage of potential memory models that either retain both memories, forget the first, or forget both. To test this, we followed a similar procedure as in memory testing. However, in this case two memories were attempted to be formed at the same time (see Section 4).

Finally, we attempted categorizing memory in the surveyed networks on the basis of species in the evolutionary strata. We considered eight categories, namely, bacteria, slime mold, yeast, plant, lower vertebrates, mammals, and general. Here, general corresponds to the networks not associated with any species but related to generalized biological processes. We found no privileged categorization of memory over classes of organisms—memory seemed distributed uniformly over all strata of life (see Figure S3). This may suggest that memory capabilities are not unique to a particular stratum, reinforcing the hypothesis that proto-cognitive capacity may be found across a variety of processes.

### 2.6. Biological Networks Have Unique Memory Profiles

We sought to test whether trainability in these networks is a mathematically inherent feature of network dynamics or if biological networks are unique in the space of possible networks with respect to the memories they exhibit. To this end, we created 500 random synthetic biology networks for comparison (see Section 4). Each network created has a network size of 10, equal to both the mean and median size of the surveyed biological networks. We also controlled for other network measures when creating the random models, namely, number of nodes, number of edges, indegree, outdegree, betweenness, PageRank, and hubness, such that the values of these measures fell within a similar distribution of the biomodels. A comparison of 35 randomly selected random models to 35 biomodels used can be found in Figure S10, showing that they represent statistically very similar kinds of networks to the biological ones. We tested three aspects of the memory profiles across both biological and random networks to search for potential unique properties among biological networks that resulted from selection in the biosphere.

**Figure 7:**
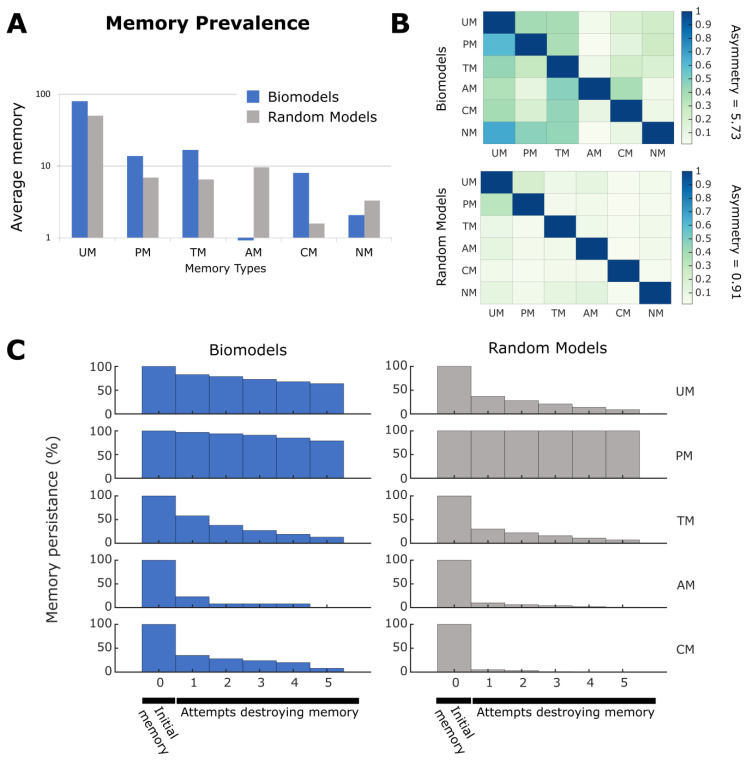
**Biological models have unique memory properties as compared to random models.** (**A**) The overall memory prevalence across all 35 biological models and 2000 random models. All random networks are of network size 10 and having average connectivity of 40%, the same as network size and connectivity average found in all biological models. The x axis are different memory phenotypes tested and the y axis the percentage of potential memory models that demonstrated the specific phenotype. In all cases other than associative memory (AM), biological models showed more memory phenotypes than random networks. (**B**) The correlation matrixes for both biological and random models. The x axis and y axis are both memory phenotypes and the squares at their intersection report the mean correlation between the two memory phenotypes across all models. Next to each is the associated asymmetry measure, i.e., how asymmetrical these matrices are. (**C**) The memory persistence for both biological and random models as a function of number of edge perturbations to the network. Each perturbation both deleted and added a random edge to the network. The y axis reports the percentage of memory models that continue to show the memory phenotype after perturbation. For this, we considered only 26 biological models (due to size constraints in edge deletion) and 500 random models, with connectivity average (40%) and larger network size (14 nodes).

First, we checked the prevalence of each type of memory in biological vs. random networks, as it may be that certain memories are more inherent to network dynamics themselves, rather than something developed through evolution and development. To do this, we computed the average occurrence for a memory type in both biological networks and the random networks. We observed for most memory types, there is an increase in the prevalence of memory in biological networks over random networks. Interestingly, the average number of no memory cases (no memory or trivial S-R relationships) is much larger in random networks, providing an important null baseline. Second, we examined if any type of memory is indicative of other memory types, i.e., if memories are correlated. We calculated the correlation matrix across all types of memories in biological networks as well as random networks and observed that memories in biological networks are more correlated to each other as compared to random networks. In addition, we computed an asymmetry measure between lower and upper half of the correlation matrices of biological and random networks to examine if there are non-trivial relations between memory types. We found that the matrix corresponding to biological network has a significantly higher of asymmetry measure (A_sym_ = 5.73) compared to that of the random models (A_sym_ = 0.91). The third aspect tested was the robustness of each type of memory obtained in both types of networks. For robustness, we first considered the initial memory capacity found in a biological network as maximal capacity. We then altered the connectivity of the network by both randomly deleting and randomly adding an edge and measure the memory capacity as compared to the maximal capacity. This altered network was again altered, and capacity measured. This was carried out five times to observe the degradation of memory as a function of the number of network alterations. We conclude that these types of memory capacity in biological networks are significantly more robust than that of random networks (Figure 7).

### 2.7. Making and Breaking Pharmacoresistance

We next sought to use these analysis methods to understand aspects of pharmacoresistance, as well as avenues for intervention. Given that our data reveal the ability of networks to store memories without structural change (purely in dynamical state), we wondered if common problems plaguing the use of drugs in biomedical contexts could be addressed by targeting this kind of process. We defined pharmacoresistance as the reduction of efficacy of a drug after consistent use, which can be modeled as habituation [111]. Note that in keeping with the generality of our analysis, this does not necessarily refer to neural habituation of psychoactive drugs but is a general phenomenon affecting pharmacological interventions in any tissue.

**Figure 8:**
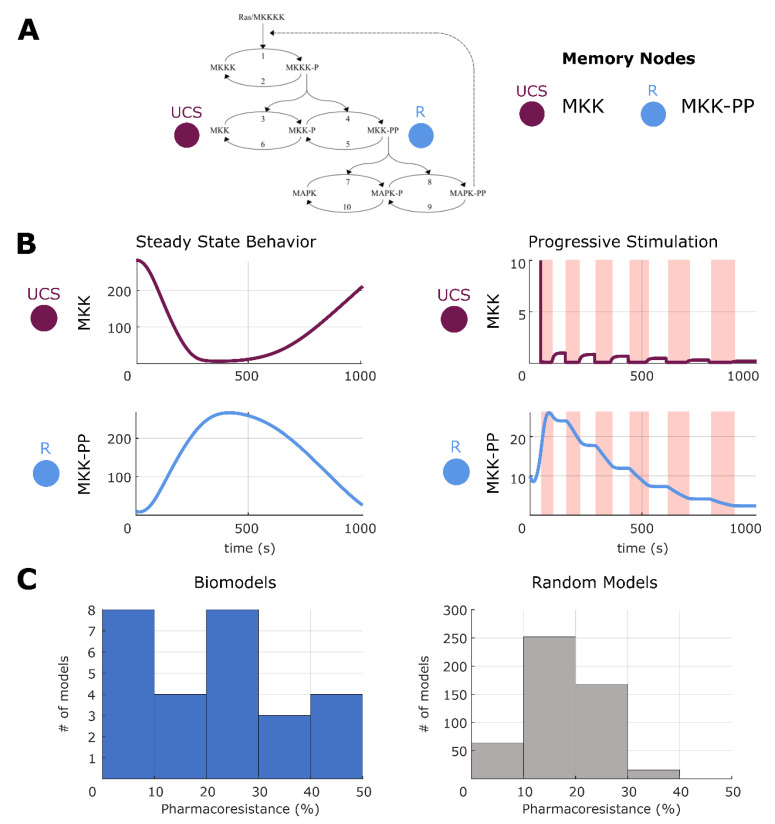
**Habituation upon repeated stimulation of UCS.** (**A**) An example of habituation in a potential memory model built from [76] that passed the pretest. (**B**) On the left, the steady state of the network with no stimulation. On the right, an example of habituation. Progressive stimulation (downregulation, red shaded area) of UCS results in less and less response from R (upregulation to downregulation). (**C**) Distribution of pharmacoresistance across all biological models and random networks. The x axis is measured as the percentage of memory models for a given biological model that show habituation, or pharmacoresistance. We performed a Wilcoxon rank sum test applicable to un-equal samples to test the similarity of the distributions and while there are differences, they are not significantly different (*p* = 0.15).

We evaluated the same models as described earlier, namely the potential memory models (all possible UCR-R combinations) built from the 35 surveyed biological models. First, we ran a pretest on each potential memory model to assess if the model demonstrated habituation, and those that did not are removed from the analysis. This pretest consisted of stimulating the given UCS for progressively increasing periods of time, with a relaxation period between each stimulation that also increased in the same manner and observing if habituation was observed in the given R (see Section 4). In quantitative terms, habituation is defined as decrease in expression by 50% (i.e., a ratio of 1:1.5) for successive stimulations. This habituation would be indicative of potential pharmacoresistance, as the desired response is increasingly more difficult despite increased stimulation time. For a visual example of this, see Figure 8. Note that here we did not test increasing stimulation strength (see Section 3). In the case of Figure 8, the habituation state can be seen as a hysteresis-like state wherein progressively increasing stimulations push the dynamics of the model towards an attractor-like state wherein the dynamics become damped into triviality. Simply put, and as can be seen 8B (right), the UCS never recovers to its original value and therefore cannot be stimulated as strongly which decreases the response seen on R. Hysteresis has been proposed to be a form of simple memory [112], albeit in this form a ‘unhelpful’ one in the eyes of a biological engineer. However, such dynamics may be present as an evolved damping state to protect against overstimulation. To quantify the degree of pharmacoresistance in a given biological model, we computed the percentage of UCR–R combinations that displayed habituation. Overall, we were able to test 27 out of the 35 models (77%, see Section 4) and at least one instance of habituation was found for each model, with the distribution of pharmacoresistance across all potential models ranging between 1–50% (see Figure 8 and Figures S11–S13).

As in memory tests, we sought to assess if habituation was native to ODE networks rather than a biological phenomenon. To do this, we again tested the 500 random biological models for habituation using the same methodology as above, and we found that the distribution of pharmacoresistance had a mean between 10 and 20%. It was observed that the distribution of the biological models differs from the random distribution, notably that it is extremely unlikely that a random model showed more than 30% of potential models with demonstratable pharmacoresistance. This would hint that pharmacoresistance may be a form of network adaptation, which would indeed be more prevalent in a biological network shaped by evolutionary pressure. Overall, we conclude that the surveyed biological networks can indeed exhibit habituation to stimulation and that it is not due to network properties alone.

**Figure 9:**
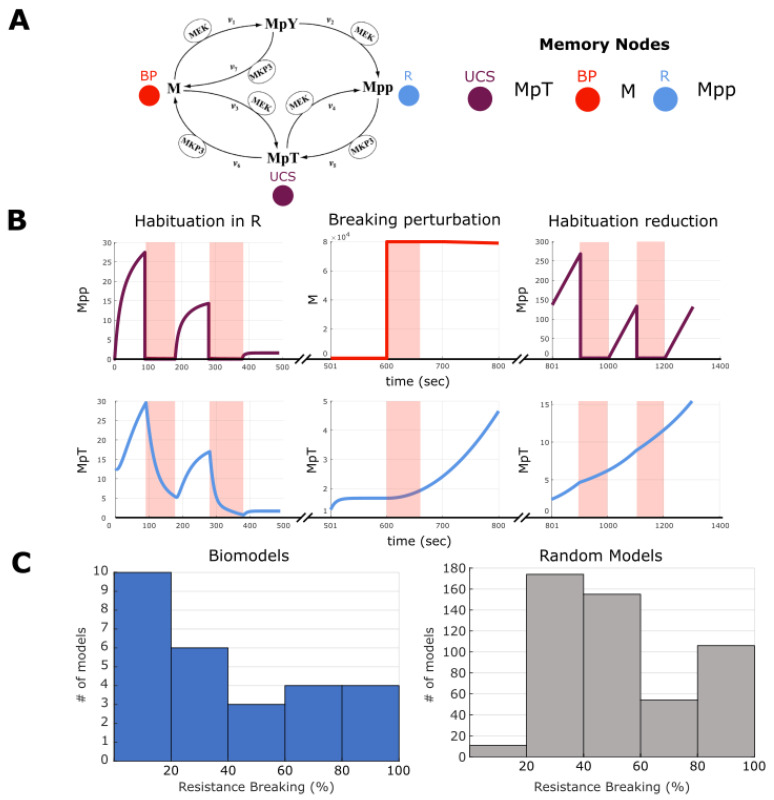
**Breaking habituation.** (**A**) An example potential memory model build from the biological model in [75] that shows both habituation to stimulation as well as breaking of that habituation through an alternative stimulation, a breaking perturbation (PB). (**B**) Time courses showing habituation (left), stimulation on the BP node (middle), and the subsequent habituation breaking (right). All graphs are a part of the same simulation and separated for visualization only. Red shaded areas indicate when a stimulation was delivered, either on the UCS or BP node. (**C**) Distribution of habituation breaking across all biological models and random networks. The x axis is measured as the percentage of memory models for a given biological model that show habituation breaking. We performed a Wilcoxon rank sum test applicable to un-equal samples to test the similarity of the distributions and they are significantly different (*p* = 0.0022).

Next, to identify therapeutic stimuli, we employed a procedure in which we search for a new ‘breaking’ stimuli on a node in the network other than the UCS and R nodes. We selected a node and delivered two stimulations followed by equivalent relaxation periods. After, we tested to see if the UCS-R relationship in the given model continued to show habituation or was broken. If R was no longer decreased by 50%, as in the pretest, this was considered a breaking of pharmacoresistance. If, however, habituation remained another trial was carried out to see if a type of ‘consolidation-like breaking’ had occurred, and if so, was also considered pharmacoresistance breaking. Out of the 27 models that showed habituation, we observed that 17 of these exhibited breaking to some degree (~63%). Again, we quantified the degree of breaking by computing the percentage of possible UCR-R pairings that show breaking. We found that, unlike baseline habituation, some models are able to always break habituation (100% of models show breaking), while overall most models can break anywhere from 20–80% (Figure 9). We also tested the random models and found that, while most models only show 20–30% of all UCR-R assignments demonstrating breaking, there is a high number of models that can always break resistance (Figure 9 and Figures S14–S16). This may be, however, due to the high fragility of random networks, where any stimulus may radically alter network states [113]. Overall, we conclude that in some biological networks, there are discoverable stimulus nodes that may serve towards breaking pharmacoresistance.

### 2.8. Making and Breaking Sensitization

**Figure 10:**
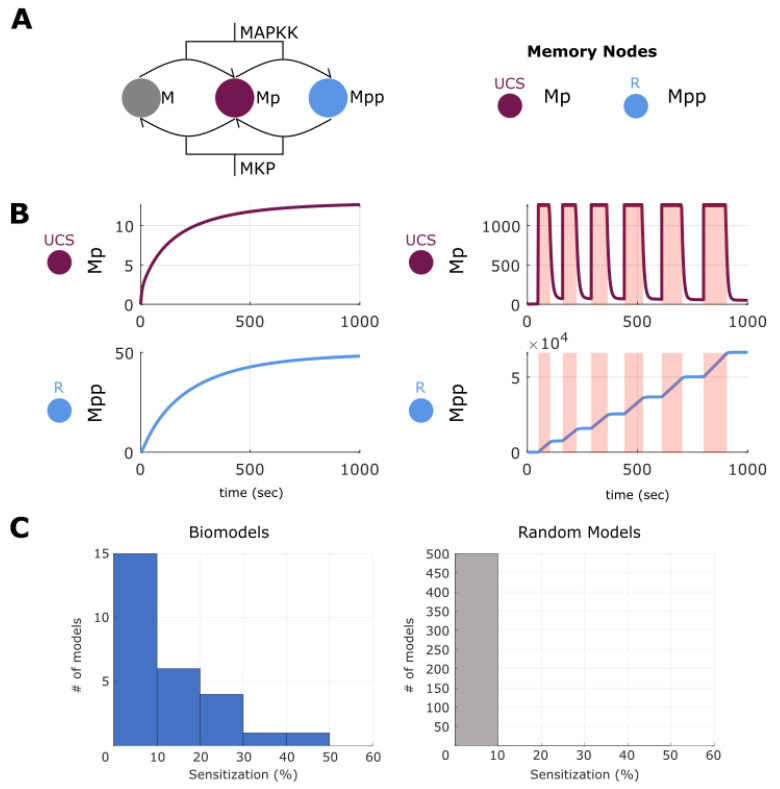
**Sensitization in biological models.** (**A**) An example of sensitization in a potential memory model built from [75] that passed the pretest. (**B**) On the left, the effects of a single stimulation—an upregulation of the UCS results in an upregulation of R. On the right, an example of sensitization. Progressive stimulation (upregulation, red shaded area) of UCS results in less and less response from R (upregulation to upregulation). (**C**) Distribution of pharmacoresistance across all biological models and random networks. The x axis is measured as the percentage of memory models for a given biological model that show sensitization. We performed a Wilcoxon rank sum test applicable to un-equal samples to test the similarity of the distributions and they are significantly different (*p* = 5.13 × 10^−70^).

Habituation is mirrored by an inverse phenomenon: sensitization. The biomedical version of this occurs when, for example, a drug can only be tolerated for a short time in a patient, due to mounting side-effects or increasingly strong tissue responses [114,115,116,117,118,119,120]. We adopt the similar pre-training as in memory and in habituation. We first checked that sensitization could be found in the surveyed ODE models. To do this we build a paradigm, exactly the same as in pharmacoresistance, wherein a stimulation was applied to a UCS node six times with following relaxation periods, with each of these epochs progressively increasing in time. We classified a change in R as sensitization if the expression level after stimulation increased by 1.5 times more than expression before (opposite of habituation). As in habituation, one can view sensitization as a hysteresis memory state—one in which increasing stimulus causes increasing response, potentially useful or harmful, depending upon the desired dynamical behavior. Using the same computational approach as before we found that again, 27 models out of the 35 surveyed had at least UCR-R variation that demonstrated this sensitization. We quantified the percentage of UCR-R variations for each model and found that the distribution ranged from 1–50%, with most models only showing few variations with sensitization. Interestingly, very few of the random models had UCS-R relations that generated sensitization (<10% of UCS-R pairings per model), indicating that this feature may be unique to biology, or that more complex random models would be needed to find such behavior (Figure 10 and Figures S17–S19).

**Figure 11:**
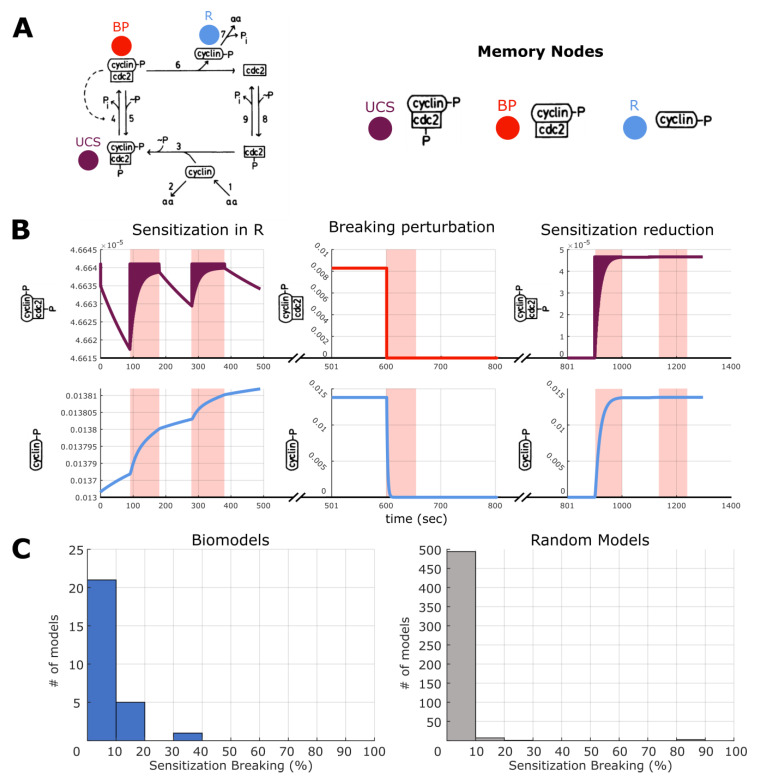
**Breaking sensitization.** (**A**) An example potential memory model build from the biological model in [77] that shows both sensitization to stimulation as well as breaking of that sensitization through an alternative stimulation, a breaking perturbation (PB). (**B**) Time courses showing habituation (left), stimulation on the BP node (middle), and the subsequent sensitization breaking (right). All graphs are a part of the same simulation and separated for visualization only. Red shaded areas indicate when a stimulation was delivered, either on the UCS or BP node. (**C**) Distribution of sensitization breaking across all biological models and random networks. The *x*-axis is measured as the percentage of memory models for a given biological model that show sensitization breaking. We performed a Wilcoxon rank sum test applicable to un-equal samples to test the similarity of the distributions and they are significantly different (*p* = 2.17 × 10^−45^).

As sensitization may pose a biomedical problem, we investigated if induced sensitization of R be overcome, or broken, as with habituation. Similar to the breaking habituation experiment, we conducted three trials. First, we applied a ‘breaking stimulation’ to a node, one other than UCS and R, allowed for relaxation, and stimulated the UCS node twice (increasing periods of stimulation and paired relaxation) and observed whether the behavior of R had broken the effects of sensitization. This was quantified by measuring if the expression rate of R no longer followed the 1:1.5 ratio of before and after stimulation (Figure 11 and Figure S20). If sensitization was not broken, a second breaking stimulation was applied to the node to see if successive stimulations affected the expression rate of R. If not, a new breaking node was chosen, and the process of stimulation and observation repeated. If no stimulation caused a breaking of sensitization, this is considered an ‘unbreakable’ sensitization. Overall, we found that 8 of 27 models with sensitization demonstrated at least one UCS-R variation that allowed for breaking. However, sensitization breaking was much rarer than for habituation, with most models having less than 5% of all variations with a break possible. Only three models showed more than 10% of all variations with breaking, however what specific dynamics are responsible for this phenomenon is outside the scope of this paper. This trend continued in the case of the 500 random models, as almost all models had less than 5% of all variations with breaking. There was one outlier however, that was able to demonstrate 100% of all variations with sensitization breaking. Overall, we conclude that while sensitization is prevalent in biological models, the breaking of sensitization is less likely than in habituation, and therefore therapeutic models may need to pursue other avenues of intervention.

## 3. Discussion

Here, we demonstrate that biological networks, such as GRNs and protein pathways, show evidence of a primitive form of memory, similar to associative and transfer memories among others, by computational evaluating a large space of stimulus–response pairings across ODE network models. This was completed as an extension of previous work on the binary GRN models, with three key extensions. First, while Boolean networks are different than ODE networks, this did not conceptually change the definition of memory, and the inclusion of three modes (upregulated, downregulated, and non-regulated) allowed for a deeper inspection of possible dynamics. Second, this study extends the possible networks from solely GRNs to protein and metabolic networks as well, pushing the conceptual limits of primitive memory in biological networks. Third, in addition to the evidence of memory, we demonstrated methodologies evaluating and breaking of drug resistance (habituation) and sensitization. With a few exceptions, we generally found memories to not be inhibited by noise (indeed, in some cases improved by noise—a fascinating aspect of biology), and to be long-lasting. In general, reading (testing) memories did not interfere with their stability, but in some cases actually increased memory (analogous to consolidation by recall, observed in neural systems). Interestingly, we found that memories are robust to a wide range of stimulus strengths (thus perhaps compatible with different levels of drug interventions achievable in vivo, for example); however, in some cases weaker stimulation is remembered better by the tissue, suggesting the need to test a diversity of dosages in applications to form a true picture of the optimal control strategy.

We found that all tested biological models had stimulation-response pairings that demonstrated some form of memory, implying that these networks may be more capable than previously thought. Importantly, not only are they more functionally capable of complex, context-specific activity, but are also more *controllable* as demonstrated via habituation and sensitization breaking. All of these results are reinforced by the finding that random networks (with similar structural properties) do not have comparable memory capabilities, nor controllability. Interestingly, since the random networks were designed to match the biological models in number of nodes, number of edges, indegree, outdegree, betweenness, PageRank, and hubness, those features are not likely to be sufficient criteria for designing (or evolving) networks with memories.

Our comparison between biological models and random ones raises the question of whether dynamic network memory could be a “generic” (inherent) property of networks [69,121], with our results indicating that certain memory phenotypes may be something that is enriched (or selected for) in biological processes. Four models demonstrated a marked increase in number of memories across the tested phenotypes: (1) a protein interaction network for the cell cycle which includes a spontaneous oscillator [77], (2) a protein interaction network for intracellular circadian rhythm generation [84], (3) mRNA/protein network for plant circadian clocks [86], and (4) gene/protein network functioning as a developmental timer which includes strong oscillatory components [91]. From this, we hypothesize that biological networks with strong oscillatory components may benefit more, from a memory perspective, to noise, similar to the case of harmonic resonance [122]. However, we note that other biomodels tested had oscillator components but did not show this increase and therefore the answer is not entirely clear.

That primitive memory is found across species is also striking, as these capabilities may also be more pervasive. The memory capability of biological models is also much more robust to edge perturbation than those of random networks, as indicated in Figure 7, perhaps due to evolutionary pressure to handle changing environments. Indeed, the pressure to adapt to variable environments is hypothesized to be one driver of basal cognition through inference [123,124,125]. The role of trainability in evolution and development is as yet unknown, but it is interesting that transcriptional [126] and other [127,128] biological signaling mechanisms are now seen as pulsatile, which affords rich opportunity for cells to apply timed “behavior shaping” stimuli to each other in vivo, as a way of exploiting the multiscale competency of biological material to evolve complex phenotypes [129]. Indeed, even despite having radically different timescales of function and memory, many studies indicate that signaling pathways can be conceptually viewed as proto-cognitive systems [130].

There are several limitations to this study. Most notably, that these networks are studied as a model in isolation, where their behavior may be different from how they would function in vivo. However, this introduces an interesting further direction to this work, exploring the testing of feasible models on the bench, to test the controllability of primitive memory in a more biological setting. This would further allow for testing for habituation and sensitization, including finding suitable biological stimulations for breaking either. Even if in a biological setting these phenomena may be harder to detect due to adaptive mechanisms present in an organism, or harder to alter, these results can be used to (1) design synthetic biology circuits with advanced capabilities [131,132], and (2) conduct studies of subcellular proto-cognitive phylogenetics, to help understand the evolutionary pressures for and against trainability in cell regulatory machinery.

Network model dynamics do not map one-to-one onto all paradigms in behavioral science, and future work will refine the definitions of key concepts in the literature to broaden existing terminology in the field of memory research or define new terms appropriate to specific kinds of systems. However, mapping such concepts across fields and across material substrates allows for the development of new hypotheses and ideas where progress has stagnated as others have also shown [133,134,135]. New frontiers in evolutionary developmental biology can be discovered by taking the origins of cognition seriously, such as primitive forms of memory, in translational studies. Here, we believe we have taken small steps towards that end, computationally exploring top-down control of deep, complex dynamics through stimulus–response pairings to enact change, rather that attempting to address symptoms such as sensitization through more traditional bottom-up approaches. One far-reaching application for this type of approach is in personalized medicine [136,137], wherein patient-specific applications could be built to reduce side effects or falling efficacy by testing possible stimulus–response pairs that meet desired criteria. This work furthers a roadmap for exploring trained drug response, or drug–drug response conditions [138,139,140,141], but on the level of GRNs and protein networks. Moreover, it suggests possible mechanisms that could help to understand unconventional memory effects in organ regeneration [142] and the persistence of memories across brain remodeling and repair [143].

The relationship between canonical synaptic memory plasticity mechanisms [144], novel molecular substrates such as RNA [145,146] and protein [147,148], and molecular networks’ dynamical systems memories is as yet unknown. However, the study of trainability and memory effects across biological organization is sure to provide rich fodder for fundamental science and applied biomedical/bioengineering applications for the future.

## 4. Materials and Methods

### 4.1. Biological Protein/Genetic Models

We downloaded 35 biological network models comprising protein/gene/metabolites (nodes) and their mutual reactions (edges) from the BioModels website [97,98,99]. Each network is modelled on chemical rate law [149] based ordinary differential equations (ODE) [150,151,152,153] and associated with a peer reviewed article on biologically tested experiments. We list all models in Table S1 mentioning the identification number of a model called “BioModels Id” provided by the website and the linked research paper. For each model, we obtain an OCTAVE (.m) file from Biomodels website and converted into corresponding $MATLAB^{\left(TM\right)}$ 2021a file with minor modification, which when executed provides the numerical solution of the system of ODEs and provides the trajectories of all species over time (t). This Matlab file (1) initializes the species with the appropriate biologically approved floating point values, (2) defines the time span over which derivatives are being calculated, and (3) uses a Matlab ODE solver [154,155] (ode23tb) which calls a function f() that takes integration tolerance level ‘Abstol’ of 1e-3, current values of x and t as arguments, and returns the time derivative  dxdt, i.e., the rate of change of the chemical species (x) over time (t) (an n tuple vector: ($dx_{1}/dt$, $dx_{2}/dt$, …, $dx_{n}/dt))$. This function (a) declares and initializes parameters (catalysts etc.) affecting the reactions, (b) defines reactions as a function of different species ($x_{i},\;x_{j},\;etc$), parameters and constant terms), and (c) define derivatives ($dx_{i}/dt$, i=(1, 2,…,n)) as a function of reactions, parameters and constants.

### 4.2. Random Synthetic Biology Models for Comparison

We created 500 synthetic biology networks to compare with the biological networks on their memory capacity, pharmacoresistance, and sensitization. Though our biological models include protein as well as genetic networks, the random models are based on synthetic gene networks. This model is based on the well-known gene circuit method [156] which constitutes an ODE equation representing genetic regulations, diffusion through cellular membrane from environment and decay of genetic materials (see Equation (1)).

(1) Time derevative=Genetic regulation+Diffusion though cell membrane Gene decay 

This equation was discretized, enhanced later, and used for model learning [157,158,159] (see Equation (2)). Here, the diffusion part [156] is excluded for the unavailability of diffusion information from extra-cellular environment through cellular membrane. Equation (2) takes a set of parameters (see Table 1) representing the model and the current expression $e_{i}\left(t\right)$ of gene i and calculates its expression at time $e_{i}\left(t+\Delta t\right)$.

(2) ei(t+Δt)= ΔtԎif(∑j=1nωijej+βi)+(1−ΔtԎi)ei(t)

Table 1 shows the set of parameters of a 3-node model. There are 3 types of parameters: (1) weight parameter ($\omega_{ij}$) represents the strength of a genetic regulation between gene i and j (n×n parameters), (2) basal expression parameter ($\beta_{i}$) represents the expression of gene i (n×1 parameters) without having regulation of other genes, and (3) time parameter (${Ԏ}_{i}$) represents the elapsed time taken by gene i (n×1 parameters) between being regulated and getting activated to regulate other genes in turn.

To create an n node random model, we generated an n×(n+2) random parameter set while each parameter belongs to the literature [157,158] specified range of the parameter (i.e., the range of $\omega_{i,j}$ = −30:30; $b_{i}=-10:10;\;{Ԏ}_{i}=1:15$). We designed this to be the random model because it is (1) based on ODEs, (2) easy to create, (3) easy to obtain time behavior by simulating the node specific equation set (see Equations (1), (2)), and (4) easy to test for memory and other biomedical properties. For a comparison of measures from both bio- and random networks, see Figure S10.

### 4.3. Memory Evaluation

We conducted memory evaluation on both biological and random models. Here, memory is defined mainly as stimuli–response effect. To describe memory evaluation, we mention a ‘stimuli–response combination’ by which we mean a combination of 3 nodes out of all possible combinations ($P_{n}^{3}$) of an n-node model. The combination comprises the unconditioned stimulus (UCS) and neutral/conditioned stimulus (NS/CS), and the response R. UCS unconditionally regulates R, i.e., when UCS is stimulated, R is also regulated. The NS does not regulate R normally and we transformed NS to CS through appropriate training procedures. As in ODE-based biological and random models, expression value of a biological entity is defined as a floating-point quantity, we can up-stimulate (increase the value to some extent) or down-stimulate (decrease the value to some extent) the quantity. Similarly, R can also be upregulated or downregulated depending upon whether ST enhances or represses its value. We consider all four possibilities in memory evaluation—(1) up-stimulated ST upregulates R, (2) up-stimulated ST down regulates R, (3) down-stimulated ST upregulates R, and (4) down-stimulated ST down regulates R (See Figure 2).

For memory evaluation of a biological model, we modified the model definition (.m file) by separating the initialization part and shaped it in the form of a function. We also separated the derivative calculation function f(t, x) from the .m file, where t represents the time steps and x represents simulations of all biological entities over t. We used the ODE solver to obtain the normal time course of each node (a chemical species) in the model over the time span (0 to 100) with the step size (h) of 0.01 and called this the ‘relax’ phase of the model. Initially, we called the ODE solver 2500 times to relax the model sufficiently (250001 time points including $t_{0}$) and obtained the maximum and minimum of each node of the model over the time course. During up stimulation of ST, we scaled up the expression value of ST to $\left(e_{ST}^{max}*100\right)$ and clamped it to a stipulated period. Through down-stimulation, we scaled down the expression of ST to $\left(e_{ST}^{min}/100\right)$ and clamped it through a period. Here, $e_{ST}^{max}$ and $e_{ST}^{min}$ are maximum and minimum expression levels of ST over the initial long relax period. It is to be noted that Matlab ODE solver does not allow to manipulate the time trajectory of a variable during simulation. To get over the problem, we called ODE solver for a minimum allowable time (10), followed by setting up/down the scale value of ST and continued the process through 5000 steps to obtain an overall constant and scaled expression level of ST during its stimulation. Through stimulations we called R upregulated if the mean expression level of R during stimulation was twice that during the preceding relaxation. Similarly, through down-stimulation of ST, if mean of R during stimulation was half of mean of R during preceding relaxation, we called R as downregulated. Before going into memory evaluation, for each node of the model treated as R, we determine which other nodes are UCS/NS of R and what state (up/down) each of the stimuli persists during examination and prepare appropriate lists. We consulted this list in the memory evaluation process.

For memory evaluation of a random model, we use a randomly created parameter set of the model. Initially, we relaxed the model sufficiently by simulating (using output function of each node specified in Equation (2)) over 2500 discrete time step with interval 0.01. Apart from the initial difference, all other procedures for memory evaluation of random model resemble that of biological model. We defined five kinds of memories applied on a valid combination of [UCS, NS, R] and devised their evaluation procedures as described below:

(1) UCS Based memory (UM) [65]—This type of memory is associated with the stimulation of UCS alone. During the memory evaluation, we stimulate UCS and check if R is regulated. After a delay, we halt the stimulation and observe if the R retains its regulated state. Conceptually, we are evaluating if stimulation of UCS causes a long-term (as compared to the stimulation) change in the behavior of R.
(2) Pairing memory (PM)—This type of memory is associated with the stimulation of paired UCS and NS. Here, we stimulate the paired [UCS, NS] and examine if R is regulated. If so, we relax the model and observe if R still continues retaining regulated state. Conceptually, we are evaluating whether the *paired* stimulation of UCS causes a long-term (as compared to the stimulation) change in the behavior of R.
(3) Transfer memory (TM) [74]: This memory does not demonstrate retention of the effect of stimuli on response R as in pairing, but it is based on change of behavior of the response by the stimulation of other stimuli. Here, initially we confirm UCS regulates R and NS does not regulate R. After, we stimulate UCS alone and after a short period, test to see if the NS begins regulating R. In other words, we check to see if NS becomes CS. Conceptually, we evaluate if the network shifts after the UCS stimulation, allowing R to be regulated by NS/CS.
(4) Associative memory (AM): Like TM, we are interested in transforming NS to CS. The concept of AM resembles classical conditioning [160,161]. Here, we check if conditions like UCS regulates R and NS does not regulate R is. Next, we train the model by stimulating UCS and NS simultaneously. Finally, we test if NS now started regulating R. Conceptually, the regulation of R has been associated with NS/CS through the simultaneous stimulation with UCS.
(5) Consolidation memory (CM): This memory is similar to AM, but with a temporal delay, as in classical consolidation. Here, we perform the same sequence as in AM detection, but crucially, the NS is *not* transformed into CS. However, after a relaxation period, we test again and see if NS has converted into CS (i.e., begins regulating R). Conceptually, the regulation of R has been associated with NS/CS through the simultaneous stimulation with UCS, but after a period of consolidation.

If neither of the memories are found within the combination of [UCS, NS, R], we call the case as no memory [78]. We consider all $P_{n}^{3}$ combinations for memory evaluation and calculate percentage of each individual memory (also no memory NM) described above.

### 4.4. Detection of Simultaneous Memories

For the detection of serial memories, we considered all 35 biological and 35 random models. Each random model has the same network size (number of nodes) as the corresponding (*i*-th) biological model. For testing, we used all existing UCS and AM memories of a network obtained through the memory evaluation process previously found and tested all possible combinations of paired memories for this evaluation, i.e., UM–UM, UM–AM, AM–UM, and AM–AM. For evaluation of the second memory after the first memory had been established, we considered the last state of all genes (numeric values) of the evaluation of first memory as the starting state of second memory evaluation. We then followed the previously described memory evaluation procedure for each type of memory evaluation for the biological and random models. We evaluated the portions having both memories retained, either memory over-written or catastrophic forgetting in each network (see Figure 6 and Figure 7).

For the detection of parallel memories, we considered all 35 biological and 35 random models. Each random model has the same network size (number of nodes) as the corresponding (*i*-th) biological model. We tested all existing combination of paired UM–UM, UM–AM and AM–AM in a network that had been previously found, as above. We then tested both memories in parallel, i.e., we started the evaluation with the initial state of all genes provided in the model definition (as in the memory evaluation procedure). To evaluate the UCS for the pair, we trained with both UCS1 (UCS of the first memory) and UCS2 (UCS of the second memory) and tested if the memory response, R, was present (R of both first and second memory). Similarly, to evaluate the AM in a pair of memories, we trained with UCS1-NS1 and UCS2-NS2 and checked if one or both NS had turned into CS. We evaluated the fraction having both memories retained, either memory over-written, or catastrophic forgetting in each network side by side for biological vs. random models (see Figure 5 and Figure 6).

### 4.5. Methodologies for Making and Breaking of Pharmacoresistance in ODE MODELS

Repeated use of a drug can make it less and less effective for pharmacological use. This phenomenon is known as pharmacoresistance [111,162,163,164]. Here, we use pharmacoresistance when repeated (back-to-back) onset of a stimulus, UCS, causes gradual decrement of R. For this experiment, we prepared a setup where we stimulated the UCS 6 times each followed by a relaxation period. Stimulation periods were incremental in nature and started with 5000 steps and proceeded to 10,000 steps incrementing 1000 in each stimulation period. Length of a relaxation period was also incremental and equal in length with following stimulation period. For the stimulation and relaxation, we exercised the same procedure described in memory evaluation. We expected that at each stimulation period, the mean expression level of R would go down by the ratio 11.5 as compared to the mean expression of R at the previous stimulation period. If the same trend continued over all 6 stimulations, we classified it as pharmacoresistance between a UCS–R combination. We examined each UCS with up- or down-stimulation and tested the effect on each R, as in memory.

For breaking the pharmacoresistance already established through repeated stimulation of UCS, we tested stimulation of every other node in the model (except UCS and R). For this, we applied this breaking stimulation for a maximum of 3 trials to determine if the resistance was broken. In the first trial, we considered a new stimuli ST1, we stimulated for 10,000 steps and relaxed for 10,000 steps. Next, we tested if the pharmacoresistance had gone. For this, we used 2 stimulations of UCS for 10,000 steps, each followed by a relaxation of 10,000 steps. If the average expression of R over the second stimulation went down compared to that over the first stimulation by the ratio 11.5 or below, we considered it as a breaking of pharmacoresistance. Otherwise, we proceeded for the second trial. If this breaking still did not occur, we repeated the third trial where we performed training using a new stimuli ST2. If testing failed this time to break pharmacoresistance, we classified the habituation as permanent.

### 4.6. Methodologies for Making and Breaking of Sensitization in ODE Models

Multiple exposures of a drug/external protein can cause the target response to grow stronger and stronger over time. This phenomenon is known as sensitization [115,119,165]. In our study, if repeated onset of a stimulus caused gradual increment of R, we classified it as sensitization. Like pharmacoresistance, we used 6 instances of UCS stimulation and relaxation, with incrementally increasing periods. If the average expression of R in (i+1)th stimulation period of UCS was 1.5 times or higher than the average value of R over ith stimulation period of UCS and this trend held for all 6 stimulation periods of UCS, we considered there was the creation of sensitization in R.

Similar to the 3 trials we used to break pharmacoresistance, here also we used 3 trials to break the sensitization of R. In trial 1, we used 2 stimulations with a new stimuli ST1 of 10,000 time steps (each followed by a relaxation period of 10,000 steps). Next, we conducted a testing phase by stimulating UCS twice for 10,000 time steps (with intermediate relaxations) and checked R to see if the average expression of it over a UCS stimulation did not exceed 1.5 times of its average over previous stimulation of UCS. If the condition satisfied, we considered that as a breaking of sensitization of R. If the first trial failed, we used 2 similar trials where training phase was conducted by stimulating new stimuli ST1 and ST2, respectively. If these two trials failed as well, we called the sensitization as permanent. It is to be noted that (1) ST1 and ST2 are among the nodes of the model excepting UCS and R, and (2) we examined all such nodes (for a specific UCS-R combination) in their up/down stimulation to see breaking effects of sensitization in R.

## Figures and Tables

**Table 1:** Parameter set of random synthetic networks.

	$\mathrm{Weight}\;\mathrm{Parameters}\;\omega_{ij}$			$\mathrm{Basal}\;\mathrm{Expression}\;\mathrm{Parameter}\;\beta_{i}$	$\mathrm{Time}\;\mathrm{Parameter}\;{Ԏ}_{i}$
	$G_{1}$	$G_{2}$	$G_{3}$		
$G_{1}$	$\omega_{1,1}$	$\omega_{1,2}$	$\omega_{1,3}$	$\beta_{1}$	${Ԏ}_{1}$
$G_{2}$	$\omega_{2,1}$	$\omega_{2,2}$	$\omega_{2,3}$	$\beta_{2}$	${Ԏ}_{2}$
$G_{3}$	$\omega_{3,1}$	$\omega_{3,2}$	$\omega_{3,3}$	$\beta_{3}$	${Ԏ}_{3}$

## Data Availability

Code can be found on: https://github.com/wesleypclawson/GRN_ODE. Data available on request.

## Supplementary Materials

The following supporting information can be downloaded at: https://www.mdpi.com/article/10.3390/ijms24010285/s1.
