# Supplementary material for: Learning in Transcriptional Network Models: Computational Discovery of Pathway-Level Memory and Effective Interventions
Source: Int J Mol Sci. 2022 Dec 23;24(1):285. doi: 10.3390/ijms24010285 (PMC9820177; doi:10.3390/ijms24010285)
Supplement: Supplementary file 1 [file ijms-24-00285-s001.zip › ijms-1976523-supplementary.pdf]

Supplements

1. List of biological networks from Biomodels used in this study

Table S1. A list of biological networks used. All networks are Ordinary Differential Equation (ODE) models of real biological entities such as genes/proteins/metabolites pathways in the cellular environments of biological organisms. We collected the models from a web-based repository of named Biomodels [28-30]. Searching IDs of the model given in the second column we obtained the details of a model like the parameters, the chemical species, reactions involving the species and parameters and the chemical rate law ODE equations. Each model was constructed and analyzed in the peer reviewed paper provided in 3rd column.

Network	Biomodel ID	Published Reference:
**1**	BIOMD0000000001	[1]
**2**	BIOMD0000000002	[1]
**3**	BIOMD0000000003	[2]
**4**	BIOMD0000000004	[2]
**5**	BIOMD0000000005	[3]
**6**	BIOMD0000000006	[3]
**7**	BIOMD0000000010	[4]
**8**	BIOMD0000000069	[5]
**9**	BIOMD0000000016	[6]
**10**	BIOMD0000000017	[7]
**11**	BIOMD0000000022	[8]
**12**	BIOMD0000000021	[9]
**13**	BIOMD0000000023	[10]
**14**	BIOMD0000000024	[11]
**15**	BIOMD0000000026	[12]
**16**	BIOMD0000000027	[12]
**17**	BIOMD0000000483	[13]
**18**	BIOMD0000000029	[12]
**19**	BIOMD0000000031	[12]
**20**	BIOMD0000000631	[14]
**21**	BIOMD0000000203	[15]
**22**	BIOMD0000000204	[15]
**23**	BIOMD0000000209	[16]
**24**	BIOMD0000000210	[16]
**25**	BIOMD0000000539	[17]
**26**	BIOMD0000000664	[18]
**27**	BIOMD0000000275	[19]
**28**	BIOMD0000000697	[20]
**29**	BIOMD0000000600	[21]
**30**	BIOMD0000000039	[22]
**31**	BIOMD0000000050	[23]
**32**	BIOMD0000000035	[24]
**33**	BIOMD0000000036	[25]
**34**	BIOMD0000000037	[26]
**35**	BIOMD0000000038	[27]

2. Memory-type distribution of biological networks

Table S2. A summary of the evaluated memory of all biological networks. We considered five memory types namely UCS Based Memory (UM), Pairing memory (PM), Transfer Memory (TM), Associative Memory (AM), Consolidation Memory (CM), and absence of memory specified as No Memory (NM) [31]. We evaluated each type of memory in all combinations of UCS-R pairings and calculated proportion of obtained the memory in the network. It is seen that most networks have large proportions of memory, with UM being most prominent.

Model	UM	PM	TM	AM	CM	NM
1	77.01	0.00	17.82	0.00	5.17	0.00
2	97.32	0.00	2.68	0.00	0.00	0.00
3	0.00	0.00	0.00	0.00	0.00	100.00
4	81.82	0.00	18.18	0.00	0.00	0.00
5	39.76	38.55	15.66	0.00	0.00	6.02
6	47.62	38.10	14.29	0.00	0.00	0.00
7	69.05	2.38	22.62	0.00	2.38	3.57
8	84.82	0.00	14.29	0.89	0.00	0.00
9	56.25	25.00	18.75	0.00	0.00	0.00
10	86.16	6.92	6.29	0.31	0.00	0.31
11	65.25	5.67	12.06	1.42	12.77	2.84
12	27.78	33.33	23.61	0.00	1.39	13.89
13	50.63	0.00	21.52	2.53	25.32	0.00
14	0.00	0.00	0.00	0.00	0.00	100.00
15	75.69	0.00	18.75	2.08	3.47	0.00
16	66.67	0.00	0.00	33.33	0.00	0.00
17	0.00	0.00	0.00	0.00	0.00	100.00
18	61.54	0.00	15.38	15.38	7.69	0.00
19	50.00	0.00	0.00	0.00	0.00	50.00
20	19.61	62.75	13.73	0.00	0.00	3.92
21	22.35	70.59	0.00	0.00	0.00	7.06
22	63.86	36.14	0.00	0.00	0.00	0.00
23	80.95	19.05	0.00	0.00	0.00	0.00
24	61.57	11.76	0.78	0.39	24.31	1.18
25	88.64	0.00	11.36	0.00	0.00	0.00
26	24.49	42.86	12.24	0.00	2.04	18.37
27	92.86	0.00	7.14	0.00	0.00	0.00
28	58.01	17.40	17.68	3.31	2.76	0.83
29	61.83	0.00	18.02	0.61	19.54	0.00
30	71.43	0.00	28.57	0.00	0.00	0.00
31	71.57	0.00	22.19	0.25	5.99	0.00
32	4.65	48.84	11.63	0.00	0.00	34.88
33	0.00	0.00	0.00	0.00	0.00	100.00
34	69.32	11.36	17.61	1.14	0.00	0.57
35	79.73	0.00	20.27	0.00	0.00	0.00

3. Organism-wise categorization of the memory in biological network

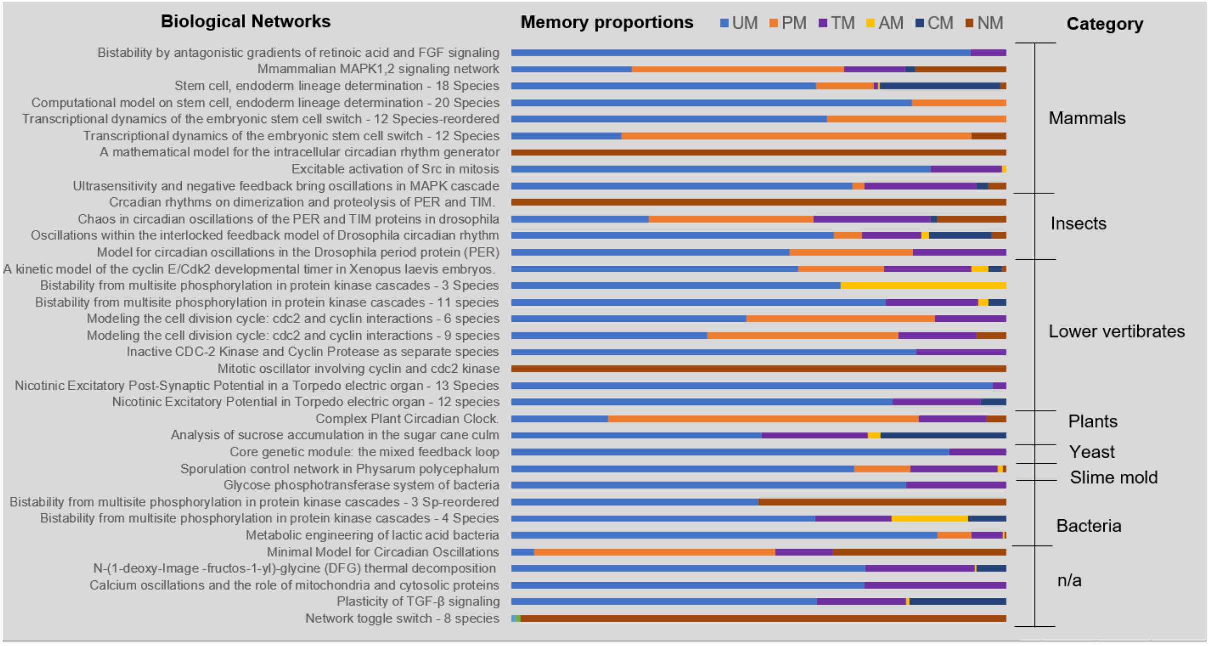

Figure S1. A depiction of our efforts of categorization of the sampled networks on the basis of organism class to gain insight on memory distributed over classes. We considered seven classes of organisms, namely bacteria, slime mold, yeast, plants, lower vertebrates, insects, and mammals. Networks are classified as “n/a” when they do not correspond to a specific organism but represent some general biological processes. All classes are seen to be similarly competent to bear cellular memory.

4. Time evolution graph demonstrating UCS Based Memory

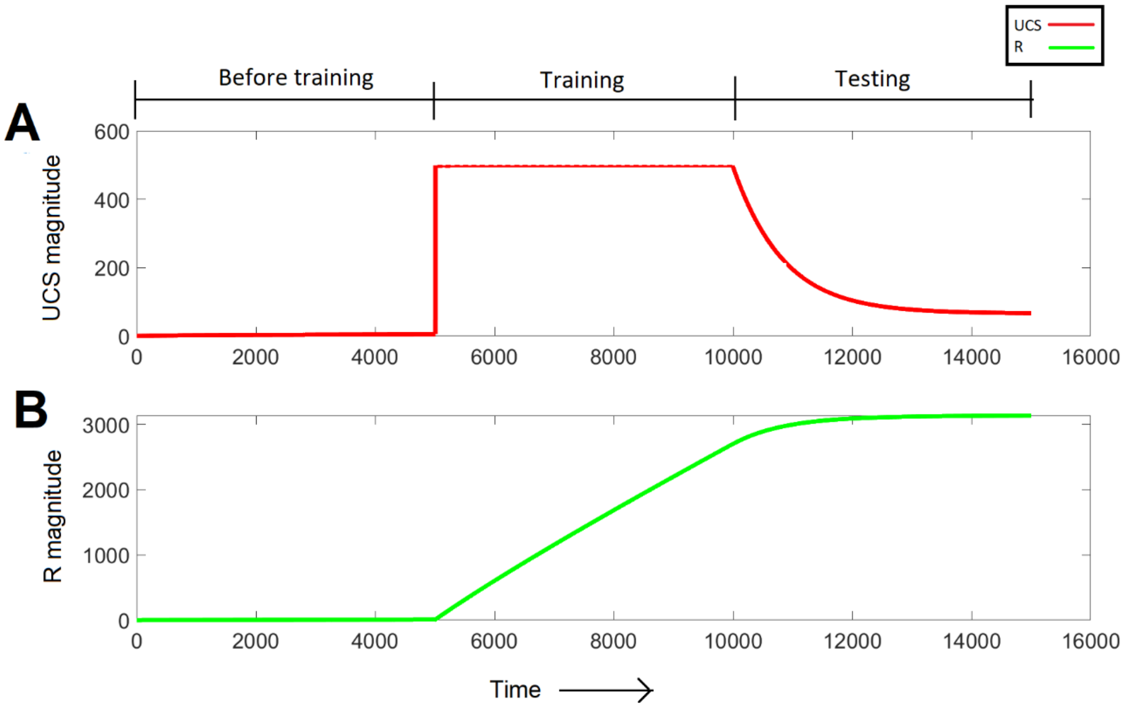

Figure S2. A time evolution graph on stimulus-response behavior in a UCS Based memory evaluation of a model collected from the Biomodel website [12]. We refer it as UCS Based memory if stimulation and clamping of UCS results in regulation of R, but when R retain its regulated state after further unclamping of UCS. The initial no intervention behavior of UCS and R can be observed in ‘before training’ region of the upper and lower panels respectively. In the testing region, we unclamp UCS to allow the network to relax. We observed that although UCS was downregulated, R remained altered in its upregulated state.

5. **Time evolution graph demonstrates Pairing Memory**

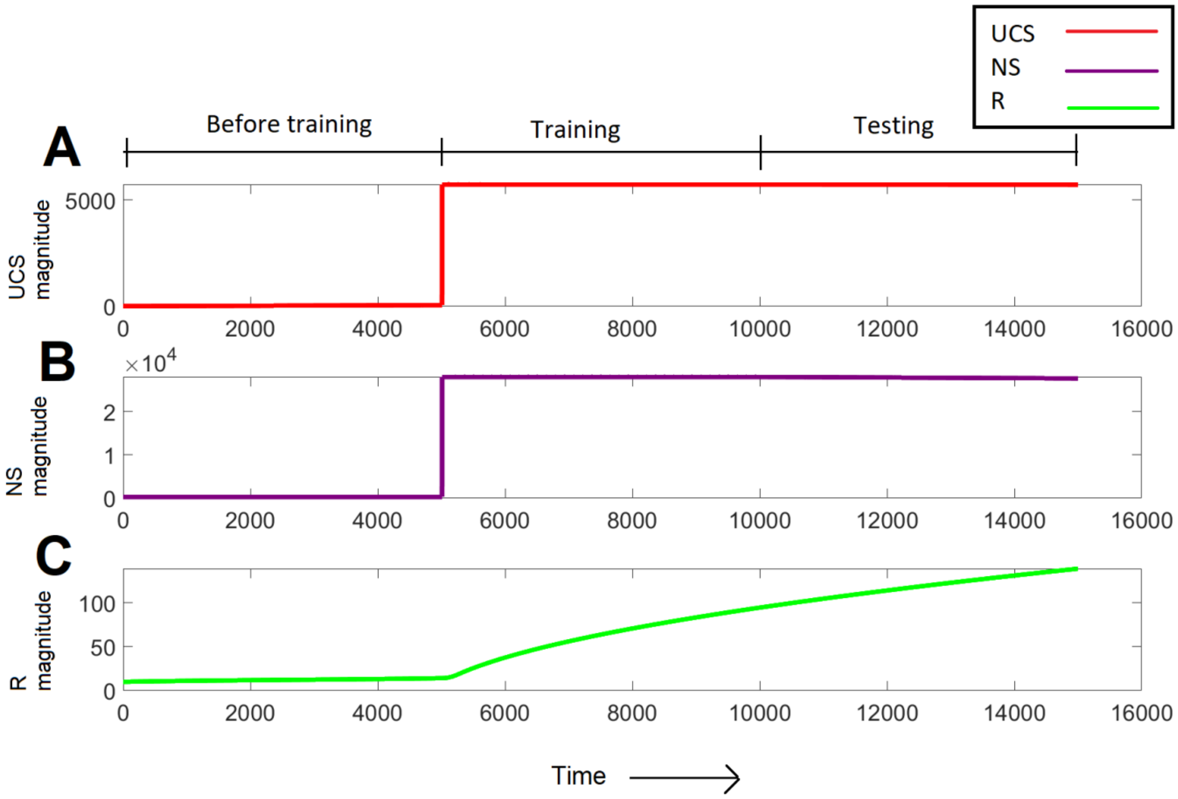

Figure S3. An example of stimuli-response behavior over time in the Pairing Memory evaluation of a biological model [4]. A pairing memory is defined when paired stimulation of UCS-NS regulates R, but R retains its regulated state after further unclamping of the stimuli. Here, UCS and NS are stimuli, and UCS upregulated R and NS had no effect. The initial steady state behavior of UCS, NS, and R are shown in the 'before training' region of the three panels respectively. We upregulated UCS and NS to 100 times of their maximum value of the before training region. As a result, R was upregulated. In testing region, we unclamp UCS and NS to relax the network. We observed that R still retains its upregulated state.

6. Time evolution graph demonstrates Transfer Memory

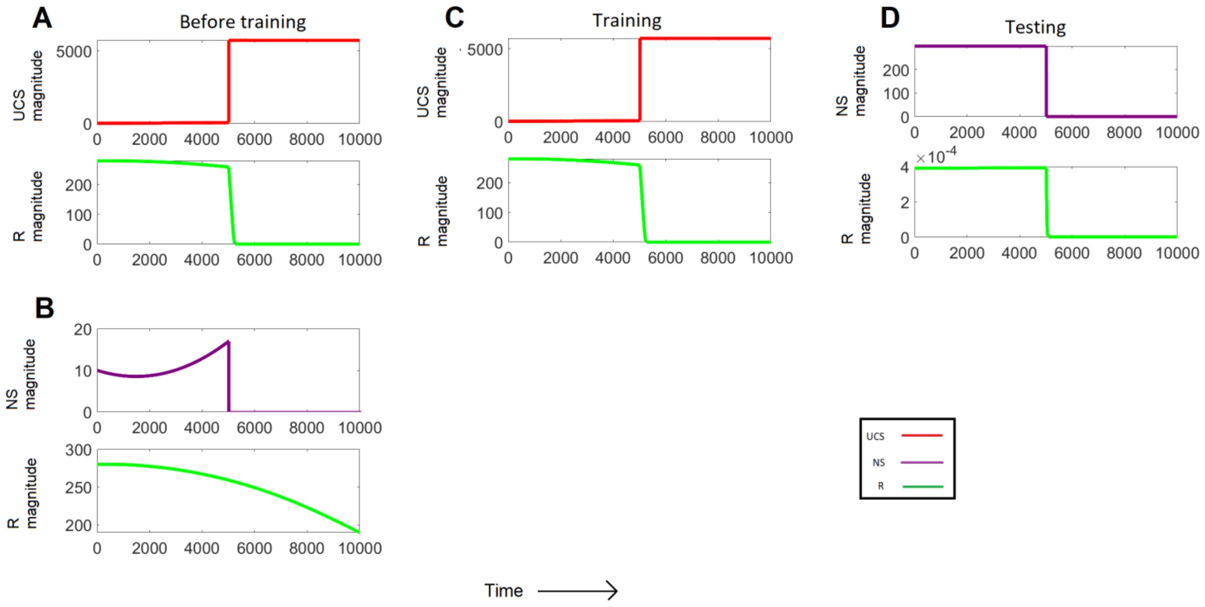

Figure S4. A time evolution graph of the stimuli-response behavior in the Transfer memory evaluation of a Biomodel [4]. UCS and NS were stimuli, and at first, the UCS downregulated R (A) while NS had no effect on R (B), which is shown in the 'before training' region plots. We first upregulated UCS to 100 times its maximum value of the 'before training' region and clamped the UCS at that value (C). As a result, R was downregulated. In testing region, we unclamped UCS to relax the network until the 5000th time step and then downregulated NS. We observed that NS had no effect on R before training but could downregulate R after training (D).

7. Time evolution graph demonstrates Consolidation Memory

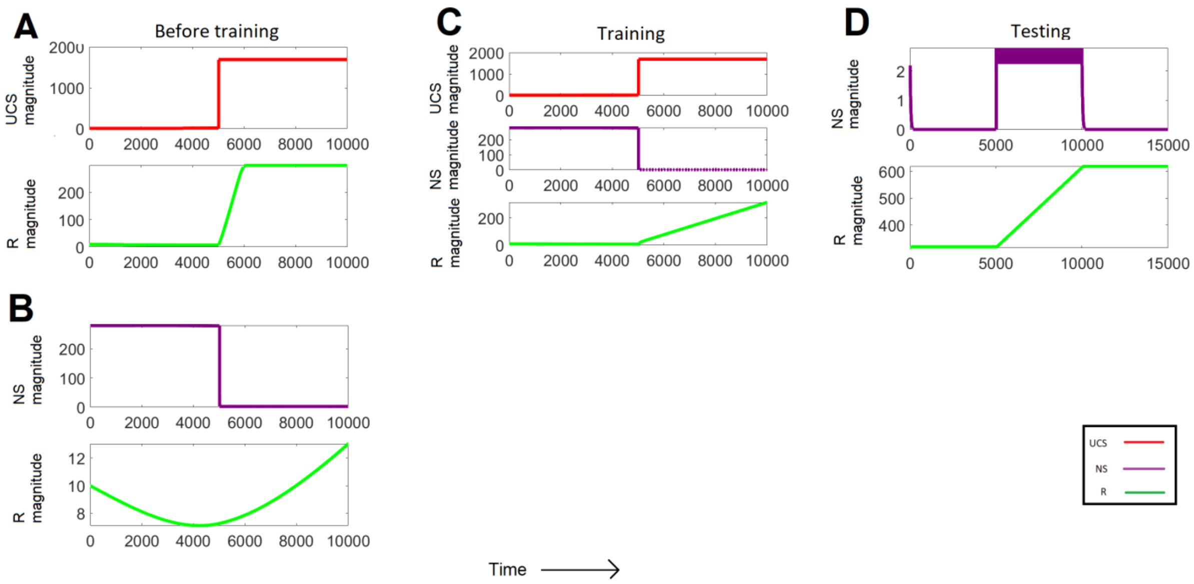

Figure S5. The stimuli-response behavior over time through a consolidation memory evaluation for the Biomodel from [4]. UCS and NS were stimuli, and UCS upregulated R (A) while NS had no long-lasting regulation over R (B), which are both shown in before training region plots. We then upregulated the UCS 100 times its maximum value while we downregulated NS to 1/100th of its minimum value (with respect to the 'Before training' period). As a result, R was upregulated (C). For testing, we unclamp both UCS and NS to relax the network and then upregulated NS after 5000th time steps. We observed that NS, which could not control R before training, could not immediately up-regulate R, but could do so after a short time period (D).

8. Memory dynamics as a function of input stimulation strengths and noise

A

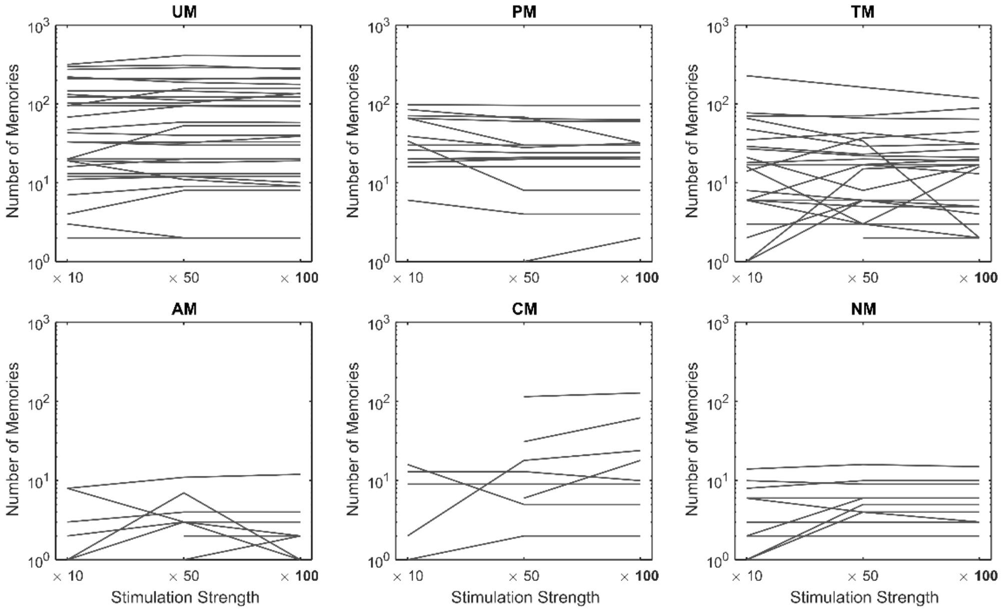

B

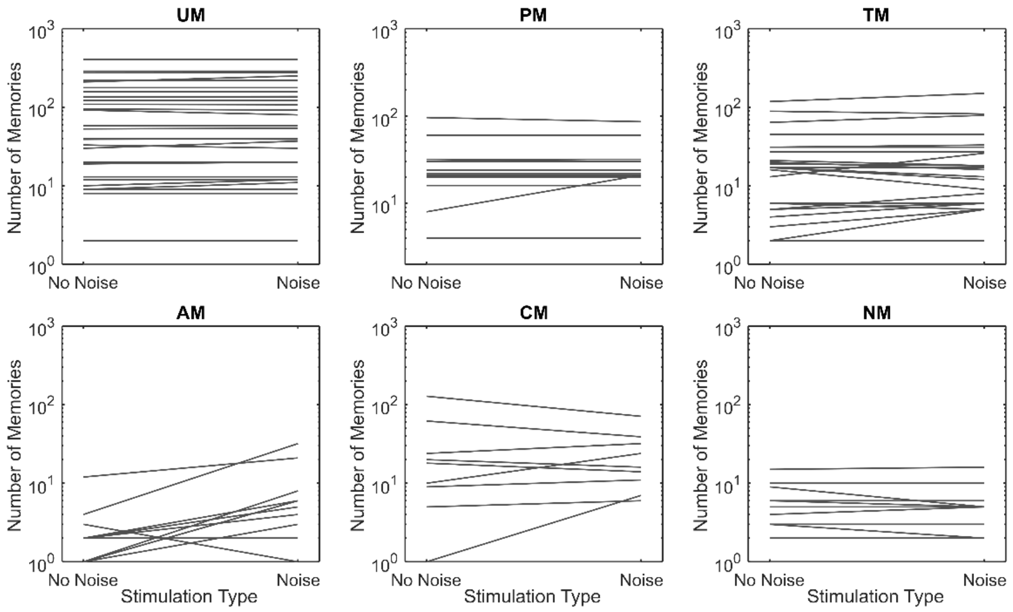

Figure S6. (A) Here we examine how the strength of the stimulation effects the formation of memories. On each graph, each line represents a biomodel across different conditions. To ensure that memories were not formed solely due to the strong stimulation (x100 of baseline is what is presented in the main text), we tested the networks on stimuli of strength 10X, 50X, and 100X of baseline behavior. We observed that reducing the strength of the stimulus 10-fold resulted in very minimal reductions in memory across the set of models (for UM, CM types); on the contrary, some of the memory types were actually increased for weaker stimuli (for PM, and TM). The networks especially affected by stimulus strength was model 10, which lost more than 50% memories when the stimulus was made weaker. However, 4 models demonstrated a 50% increase with smaller stimulations. (B) Here we examine the persistence

of memory in the presence of stochastic noise. On each graph, each line represents a biomodel across different conditions. Interestingly, while almost all models retained the memories created (83%) there was an overall 20% increase in memory in the presence of noise (averaged across models and memory phenotypes). For both graphs, if the line was reduced to 0 in a condition they were removed for visual clarity. For example, in (A) middle, bottom graph (CM) 3 models had no memory in the 10X condition and therefore the lines were not drawn.

9. Memory dynamics across various testing regimes

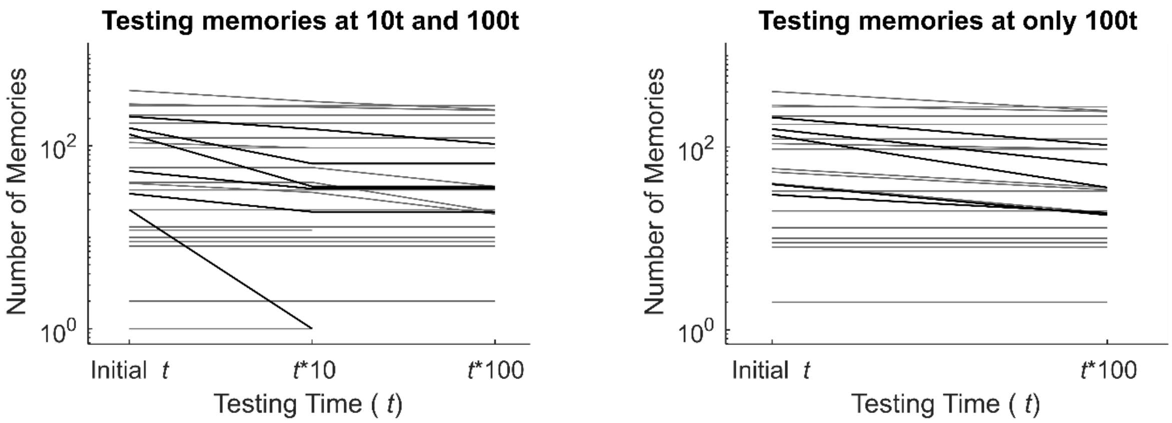

Figure S7. Here we examine how the testing regimes affect the persistence of UCS memories. For each graph, a grey line represents a biomodel across the different conditions (x-axis). Some lines have been colored black to visually highlight the specific model. (Left) Memories were tested at the original testing time after training, as presented in the main text (i.e., original data presented in main text) and compared to when testing the memories after 10X this time and then a second test at 100X this time. Out of 30 networks showing UCS memory, the a near majority did not lose significant memory capacity (defined as <50% loss) with any delay. 12 networks did lose more than 50% of memories over long timeframes, including 1, 21, and 25, each of which experienced loss of 73%, 60%, and 53% respectively (black lines). Of those losses, most occurred between 1 and 10*t timepoints but were constant after that; only eight networks lost all memories across both 10X and 100X tests. Thus, we conclude that while some networks have a limit on how long they retain training, the majority of networks can retain the bulk of their possible memories over very long time periods. (Right) To ensure that memory testing did not cause forgetting, the memories were also tested at only 100X and compared to the original testing time. The dynamics here were identical except in one case to those shown on the left, and we therefore conclude that in general, memory ‘access’ does not destroy the memory.

10. Comparison of biomodels and random models with respect to network measures

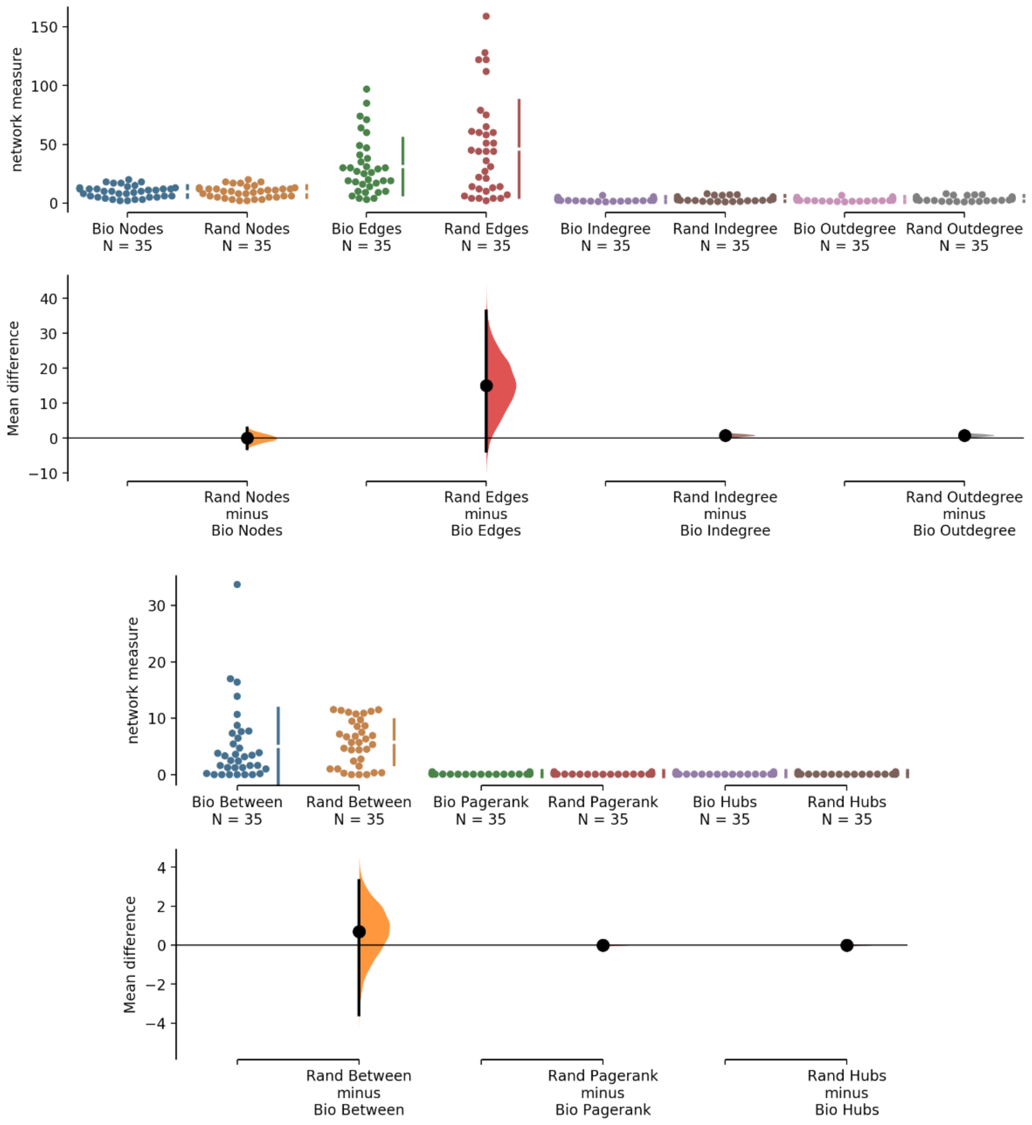

Figure S8. Ensemble statistics of biological and random model network measures were tested: number of nodes, number of edges, indegree, outdegree, betweenness, PageRank, and hubness. The raw data are plotted on the upper axes; each mean difference is plotted on the lower axes as a bootstrap sampling distribution. Mean differences are depicted as dots; 95% confidence intervals are indicated by the ends of the vertical error bars. Each dot represents a bio or random model. 5000 bootstrap samples were taken; the confidence interval is bias-corrected and accelerated.

11. Repeated onset of UCS makes habituation: example 1

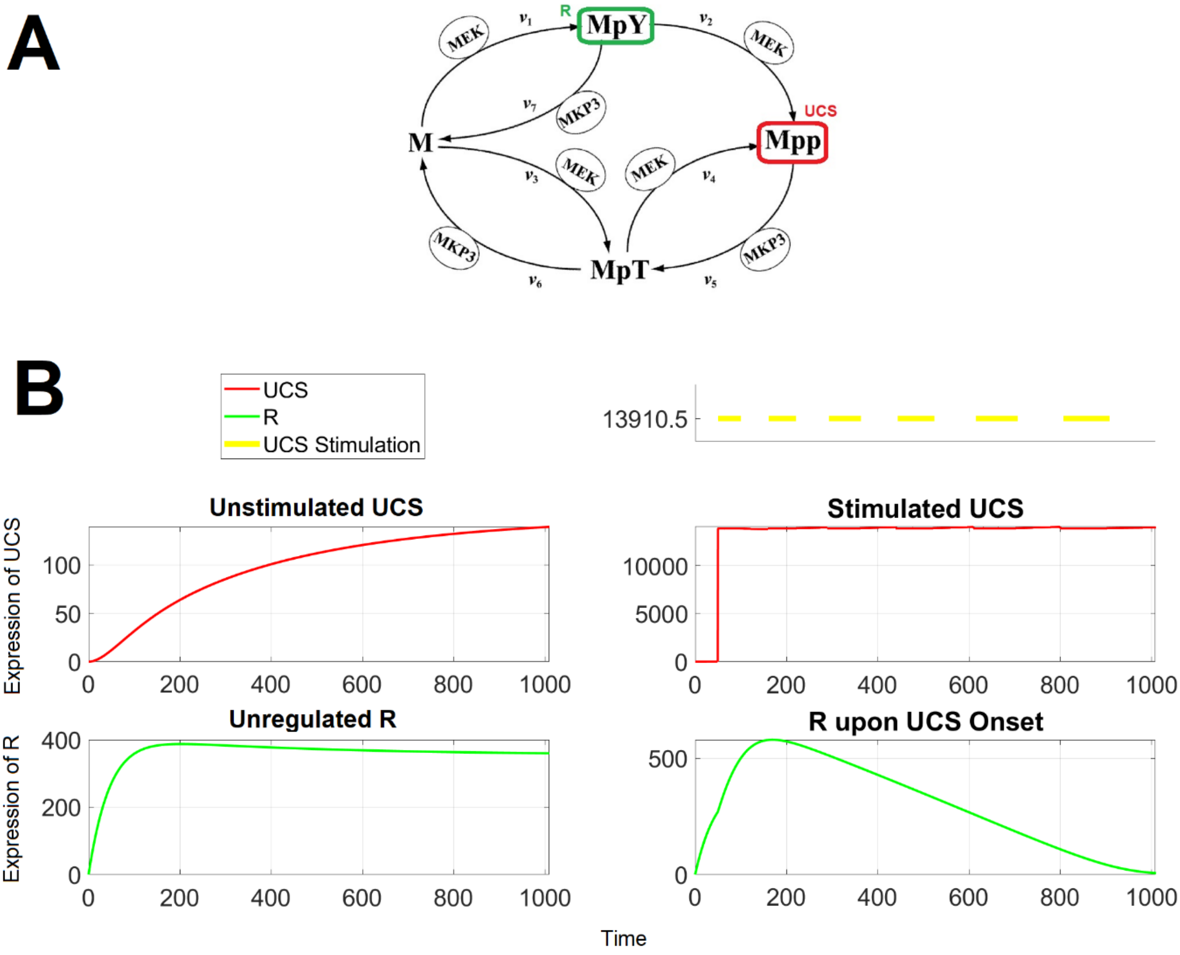

Figure S9. An example of pharmacoresistance, where the response, R, declines upon repeated stimulations of UCS within a biological network. (A) The biological network obtained from Biomodels website [12]. It is a network having four nodes (the proteins M, MpY, Mpp and MpT), their mutual reactions, and the parameters (proteins MEK and MKP3, marked beside the edges) which affect the reactions. The UCS and R are highlighted red and green, respectively. (B) Left, the steady state (unstimulated UCS and R). Right, the repeated stimulations (yellow lines above) of UCS and resultant trajectory of R. Here, we found habituation/pharmacoresistance in R through upregulation of UCS. Shown are five down stimulations with gradually increasing durations in steps of 1000 in regular intervals of 10000 time steps.

12. Repeated onset of UCS shows habituation: example 2

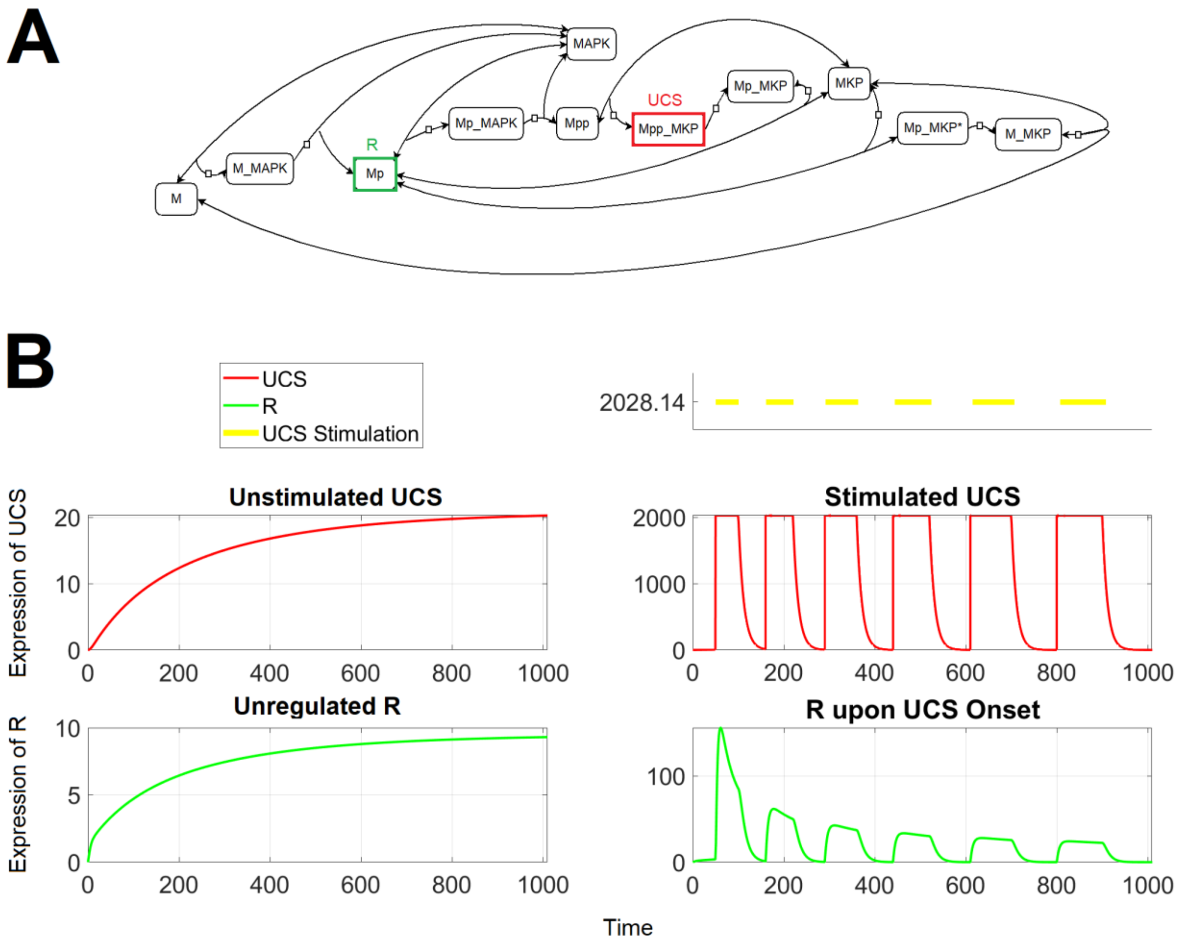

Figure S10. A second example of pharmacoresistance in R upon repeated stimulus of UCS. (A) The biological network obtained from Biomodels website [12]. It is composed of a network having eleven nodes (proteins M, M_MAPK, MP, MAPK, MP_MAPK, Mpp, Mpp_MKP, Mp_MKP, MKP, Mp_MKP*, and M_MKP) and their mutual reactions described in the model. The UCS and R are marked with red and green, respectively. (B) Left, the steady state of the model, i.e., the unstimulated UCS R. Right, the repeated stimulations (yellow lines at top) of UCS and resultant trajectory of R. Here, we found habituation/pharmacoresistance in R through upregulation of UCS. There are five up-stimulations with gradually increasing durations in steps of 1000 and at regular intervals of 10000 time steps. The trajectory of UCS shows that during every upregulation, it has equal amplitude. The maximum response is found at the first stimulation of UCS. Later, the response of R due to UCS regulations had lower and lower amplitude.

13. Repeated onset of UCS shows habituation: example 3

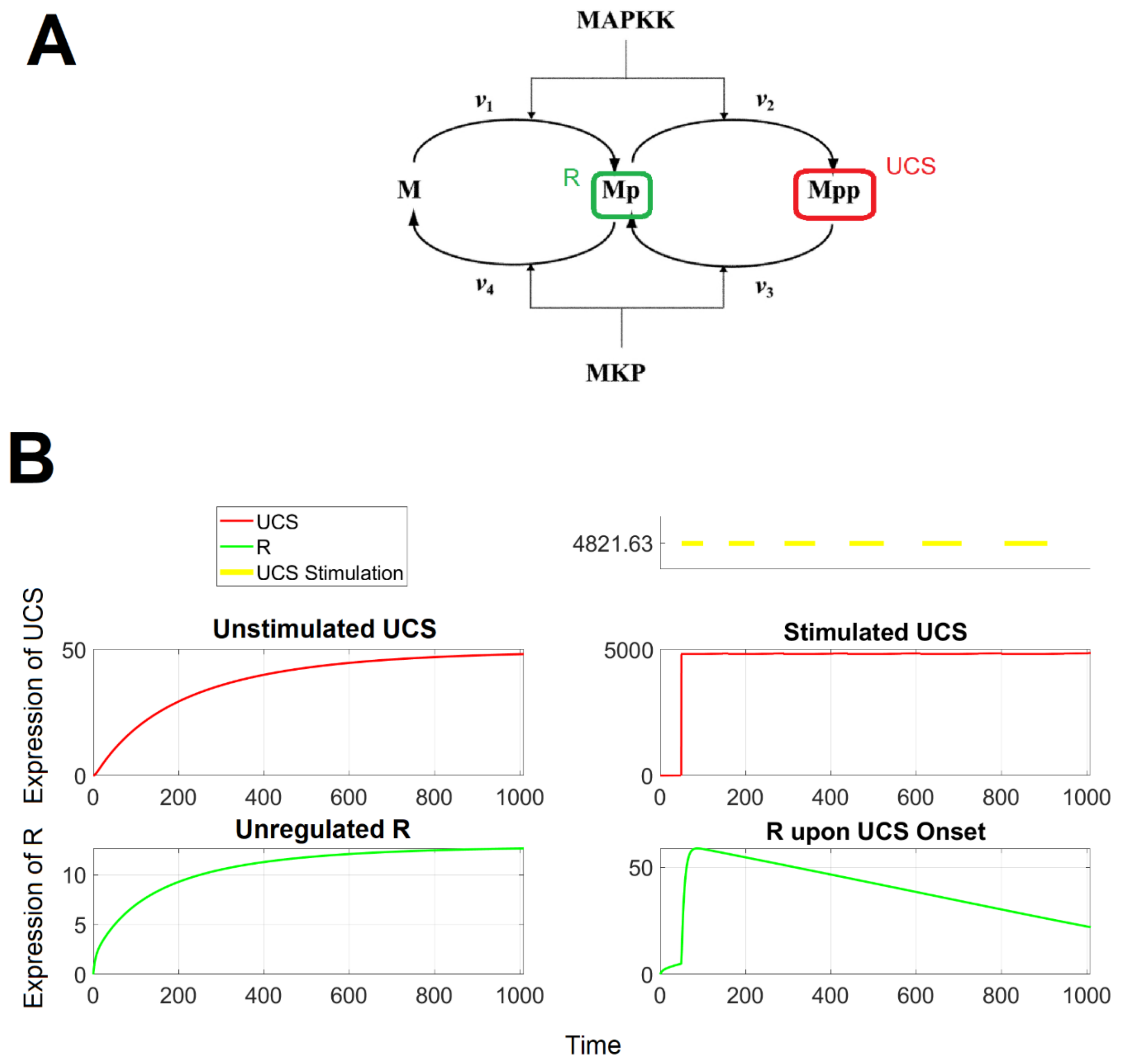

Figure S11. An example of pharmacoresistance upon repeated stimulations of UCS within a biological network. (A) The biological network obtained from Biomodels website [12]. The network has three nodes (proteins M, Mp and Mpp), their mutual reactions, and the parameters that define their relations (proteins MAPKK and MKP, marked by the edges). The UCS and R are marked with red and green, respectively. (B) Left, the steady state of the network, i.e., unstimulated UCS and R. Right, repeated stimulations (yellow lines at top) of UCS and resultant trajectory of R. Here, we found pharmacoresistance in R through upregulation of UCS. There are five upregulations with gradually increasing durations in steps of 1000 and in regular intervals of 10000 time steps.

14. Stimulation through new stimuli breaks pharmacoresistance: example 1

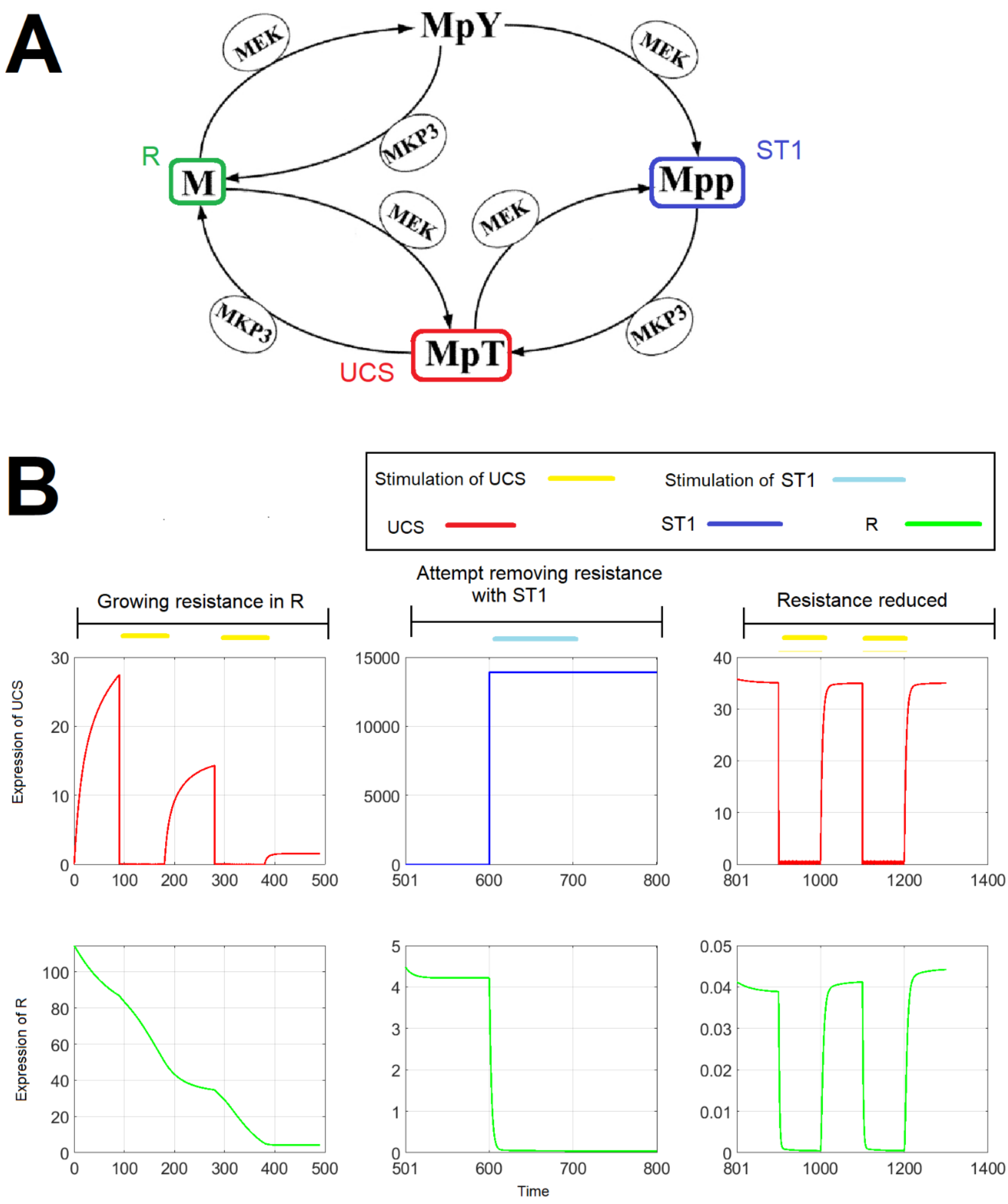

Figure S12. An example where pharmacoresistance in R is overcome by stimulation of a new stimuli. (A) The biological network taken from Biomodels website [12], which has four nodes (proteins M, MpY, Mpp and MpT), their mutual reactions, and parameters. We mark the designated R, UCS, and ST1 with green, red, and violet, respectively. (B) Left, the last two stimulations of UCS and resulting habituation in R. Here, downregulation of UCS induced pharmacoresistance in R. Middle, the attempt at breaking pharmacoresistance in R by stimulation and resulting expression of R. Right, two repetitive stimulations of UCS and resultant trajectory of R.

15. Stimulation through new stimuli breaks pharmacoresistance: example 2

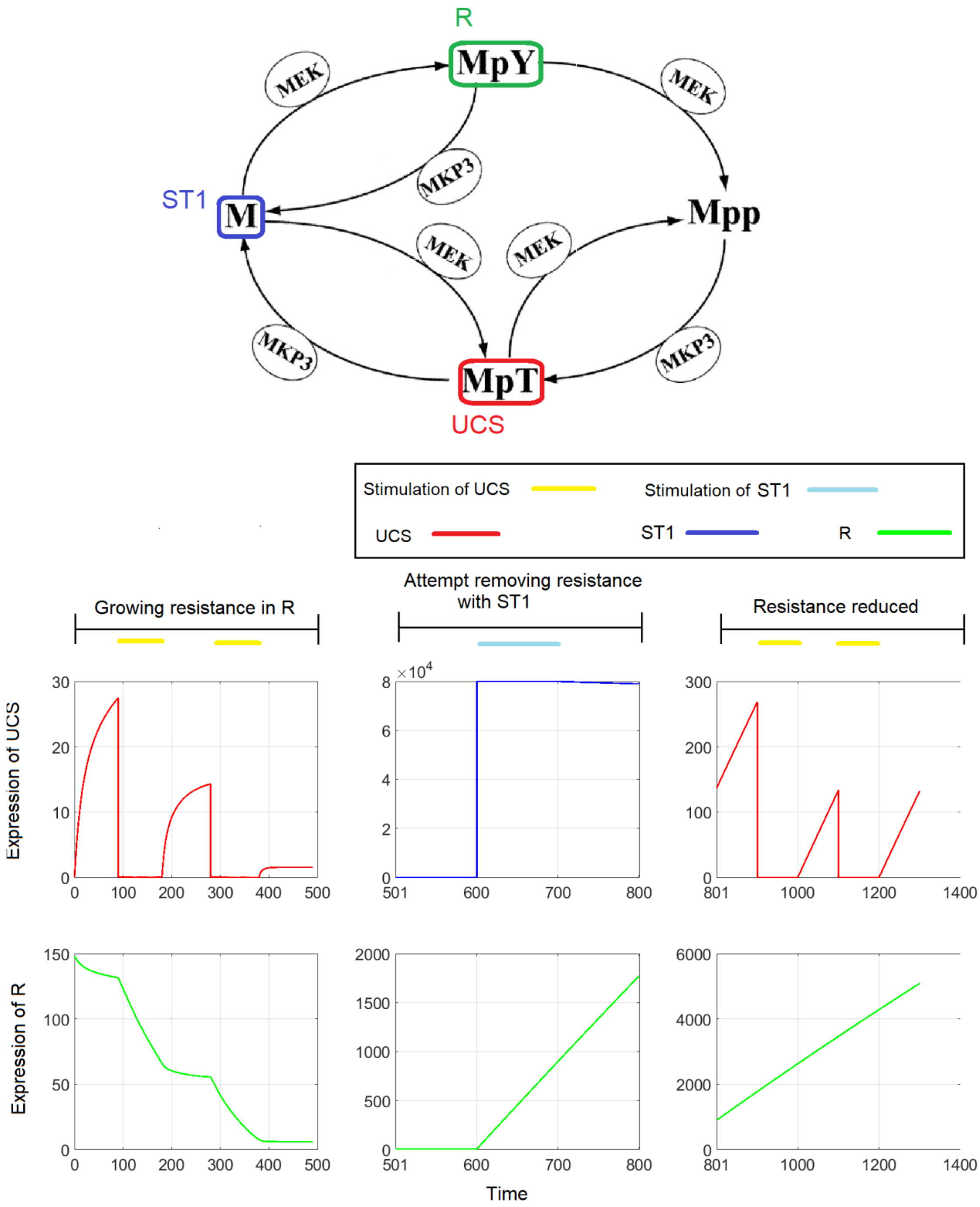

Figure S13. An example where induced pharmacoresistance in R breaks by stimulation of new stimuli. (A) The biological network taken from Biomodels website [12], composed of four nodes (proteins M, MpY, Mpp and MpT), their mutual reactions, and parameters. We mark R (MpY), UCS (MpT), and ST1 (Mpp) with green, red, and blue, respectively. (B) Left, the last two stimulations of UCS and resulting pharmacoresistance in R. Here, downregulation of UCS induced pharmacoresistance in R. Middle, the attempt of breaking pharmacoresistance in R by stimulation of ST1 and resulting expression of R. Right, the two repetitive stimulations of UCS and resultant trajectory of R.

16. Stimulation through new stimuli breaks pharmacoresistance: example 3

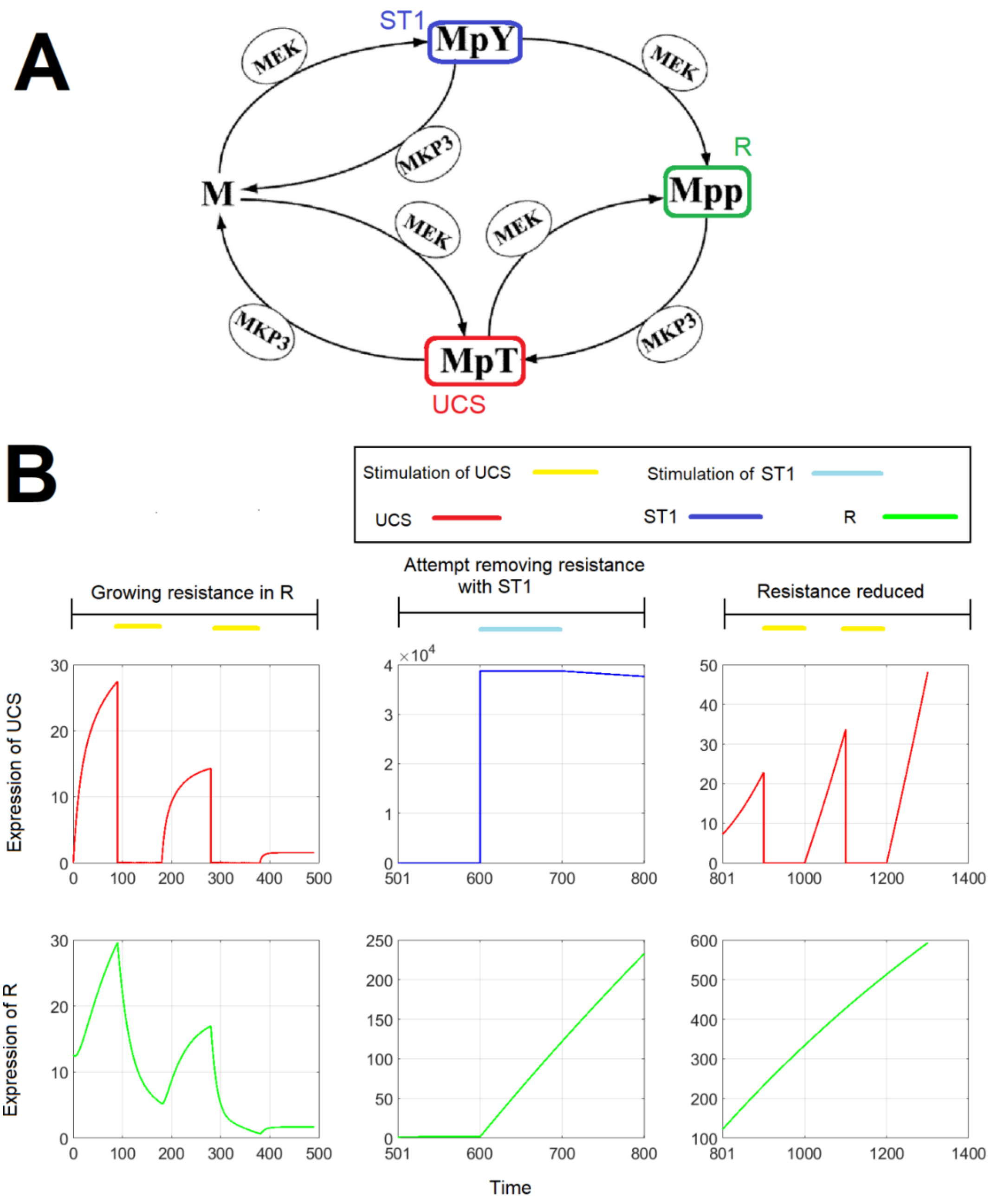

Figure S14. An example where evolved pharmacoresistance in R is broken by stimulation of new stimuli. (A) The biological network taken from Biomodels website [12], composed of four nodes (proteins M, MpY, Mpp and MpT), their mutual reactions, and parameters (proteins MEK and MKP3, marked beside the edges). We mark the designated R, UCS, and ST1 with green, red, and blue respectively. (B) Left, the last two stimulations of UCS and resulting pharmacoresistance evolved in R. Here, down stimulation of UCS developed pharmacoresistance in R. Middle, the breaking of pharmacoresistance in R by stimulation of ST1 and resulting expression of R. Right, the two repetitive stimulations of UCS and resultant trajectory of R.

17. Repeated onset of UCS shows sensitization: example 1

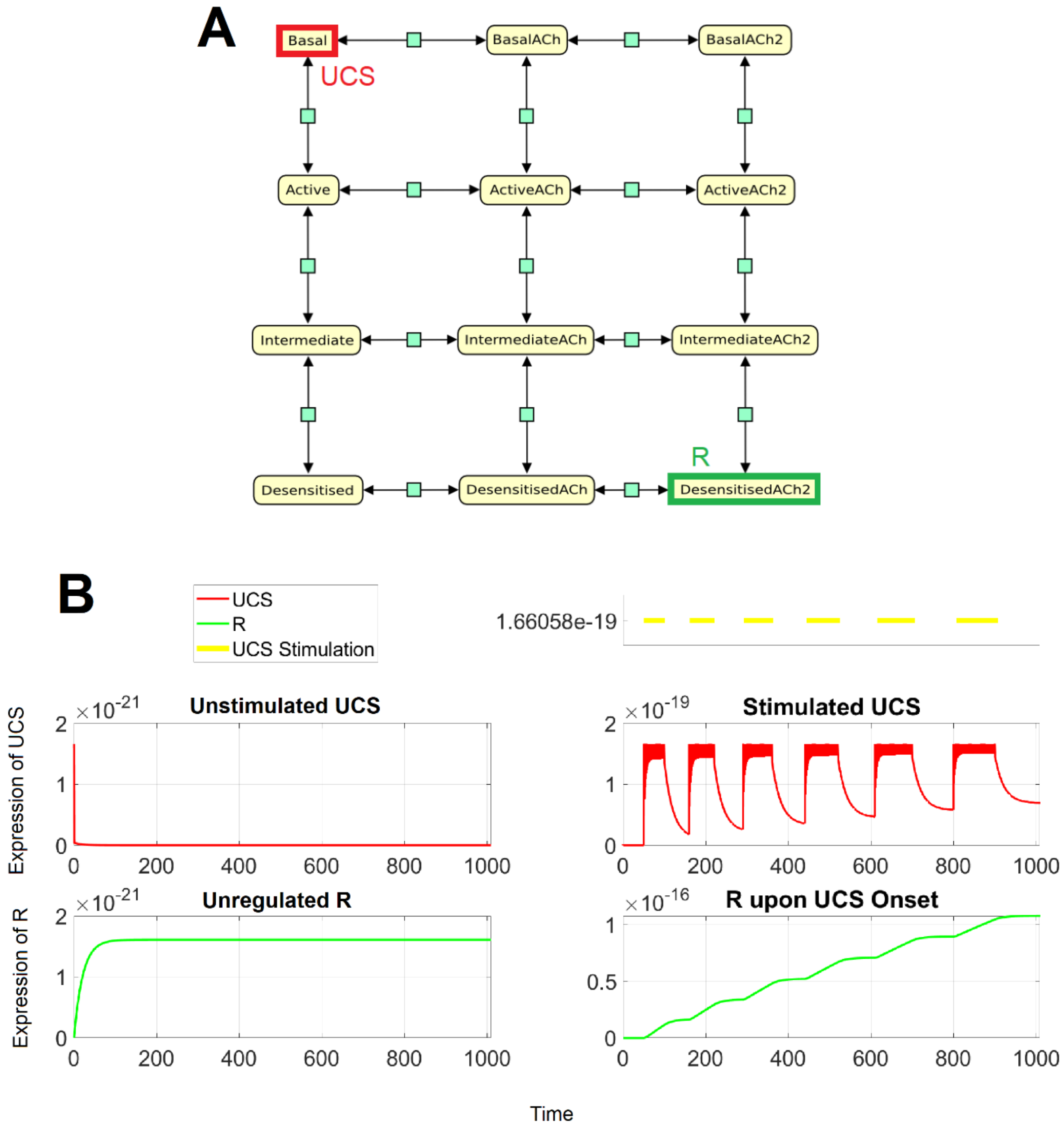

Figure S15. An of sensitization in R upon repeated stimulations of UCS. (A) The biological network obtained from Biomodels website [1], composed of twelve nodes (protein/cellular element/process: Basal, BasalAch, BasalAch2, Active, ActiveAch, ActiveAch2, Internediate, IntermediateAch, InternediateAch2, Desensitised, DesensitisedAch and DesensitisedAch2), their mutual reactions, and the parameters (proteins MEK and MKP3, marked beside the edges). We mark the UCS and R with red and green, respectively. (B) Left, the steady state of the model, i.e., the unstimulated UCS and R. Right, the repeated stimulations (yellow lines at top) of UCS and resultant trajectory of R. Here, in this case we found sensitization in R (DesensitisedAch2) through up-stimulation of UCS (Basal). There are five up-stimulations with gradually increasing durations in steps of and in regular intervals of 10000 time steps. The trajectory of UCS shows periodic increased amount through stimulation and decrease through relaxation.

18. Repeated onset of UCS shows sensitization: example 2

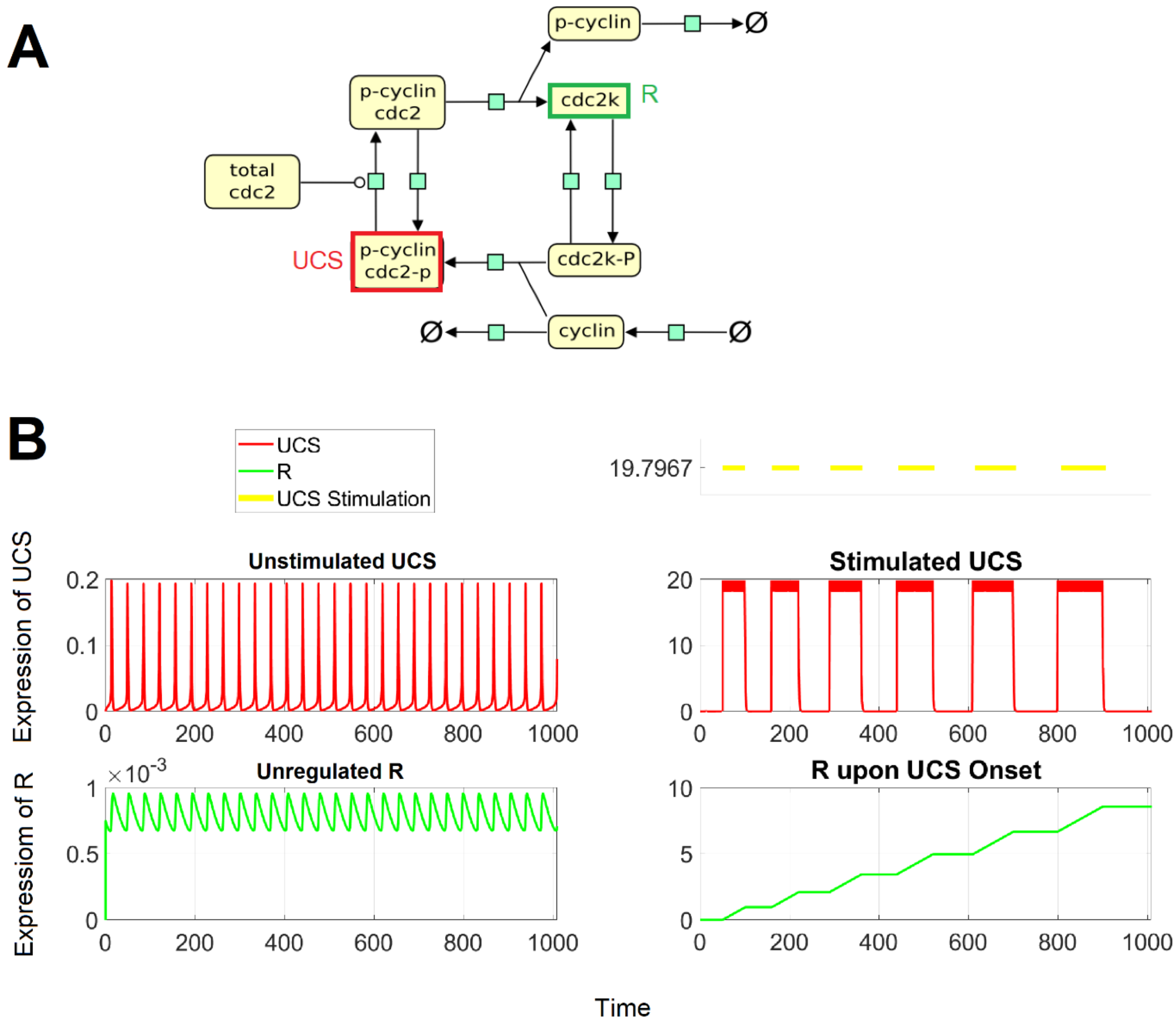

Figure S16. An example of sensitization grows in R upon repeated stimulations of UCS within a biological network. (A) The biological network obtained from Biomodels website [3], comprised of six nodes (proteins total cdc2, p-cyclin cdc2, p-cyclin cdc2-p, p-cyclin, cdc2k, cdc-2k-P and cyclin), their mutual reactions, and their parameters. We mark the UCS and R with red and green, respectively. (B) Left, the initial steady state, i.e., the unstimulated UCS and R. Right, the repeated stimulations (yellow lines at top) of UCS and the resultant trajectory of R. Here, in we found sensitization in R through up-stimulation of UCS. There are five down stimulations with gradually increasing durations in steps of 1000 and in regular intervals of 10000 time steps. The trajectory of UCS shows periodic increased response via stimulation and decreases through relaxation.

19. Repeated onset of UCS shows sensitization: example 3

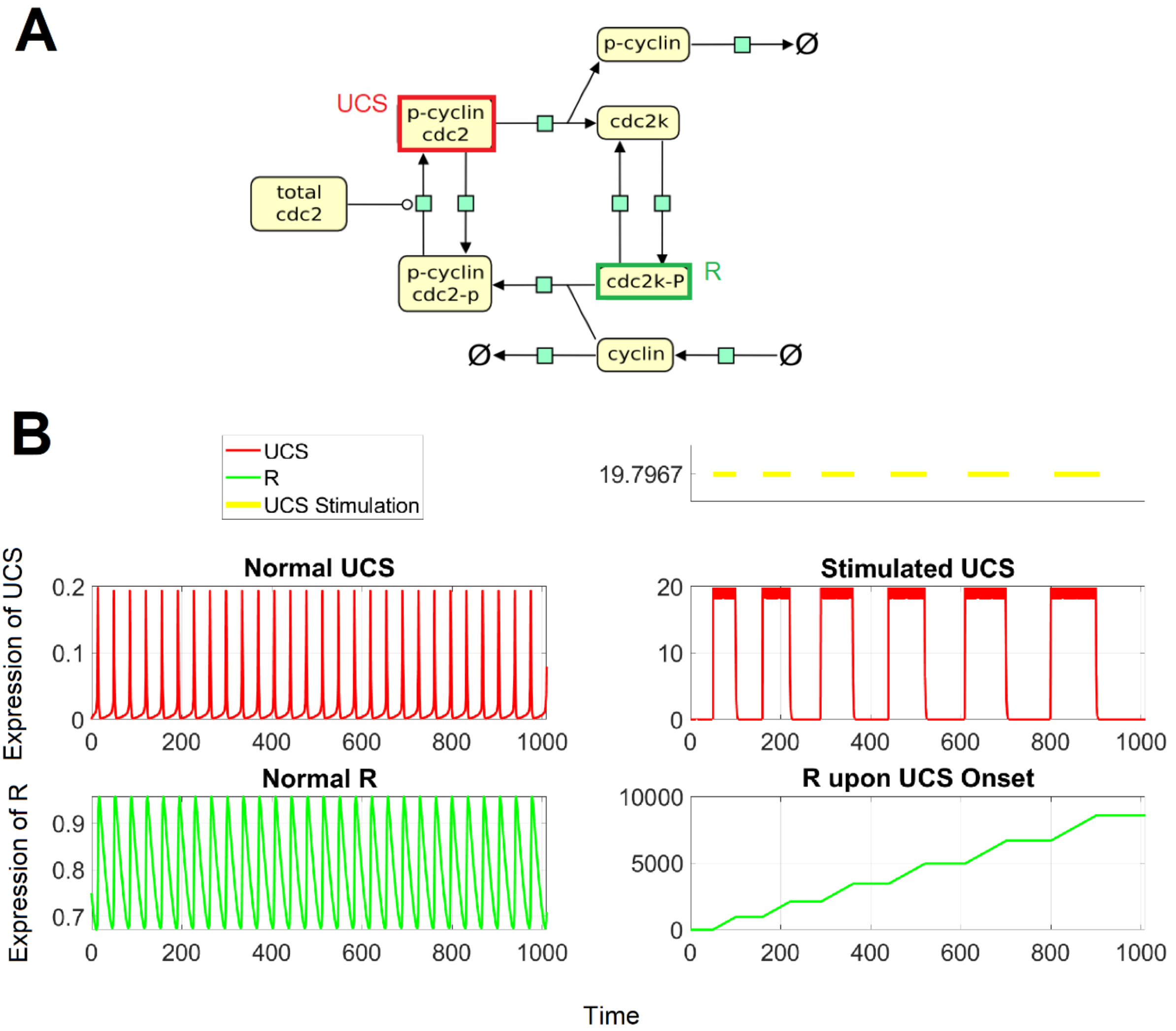

Figure S17. An example of sensitization in R upon repeated stimulations of UCS within a biological network. (A) The biological network obtained from Biomodels website [3], comprised of six nodes (proteins total cdc2, p-cyclin cdc2, p-cyclin cdc2-p, p-cyclin, cdc2k, cdc-2k-P and cyclin), their mutual reactions, and their parameters. We mark the UCS and R with red and green, respectively. (B) Left, initial steady state, i.e., the unstimulated UCS and R. Note the cyclic nature by default. Right, the repeated stimulations (yellow lines at top) of UCS and resultant trajectory of R. Here, we found sensitization in R through up-stimulation of UCS. There are five down stimulations with gradually increasing durations in steps of 1000 and in regular intervals of 10000 time steps. The trajectory of UCS shows periodic increased response through stimulation and decrease through relaxation

20. Stimulation through new stimuli breaks sensitization: example 1

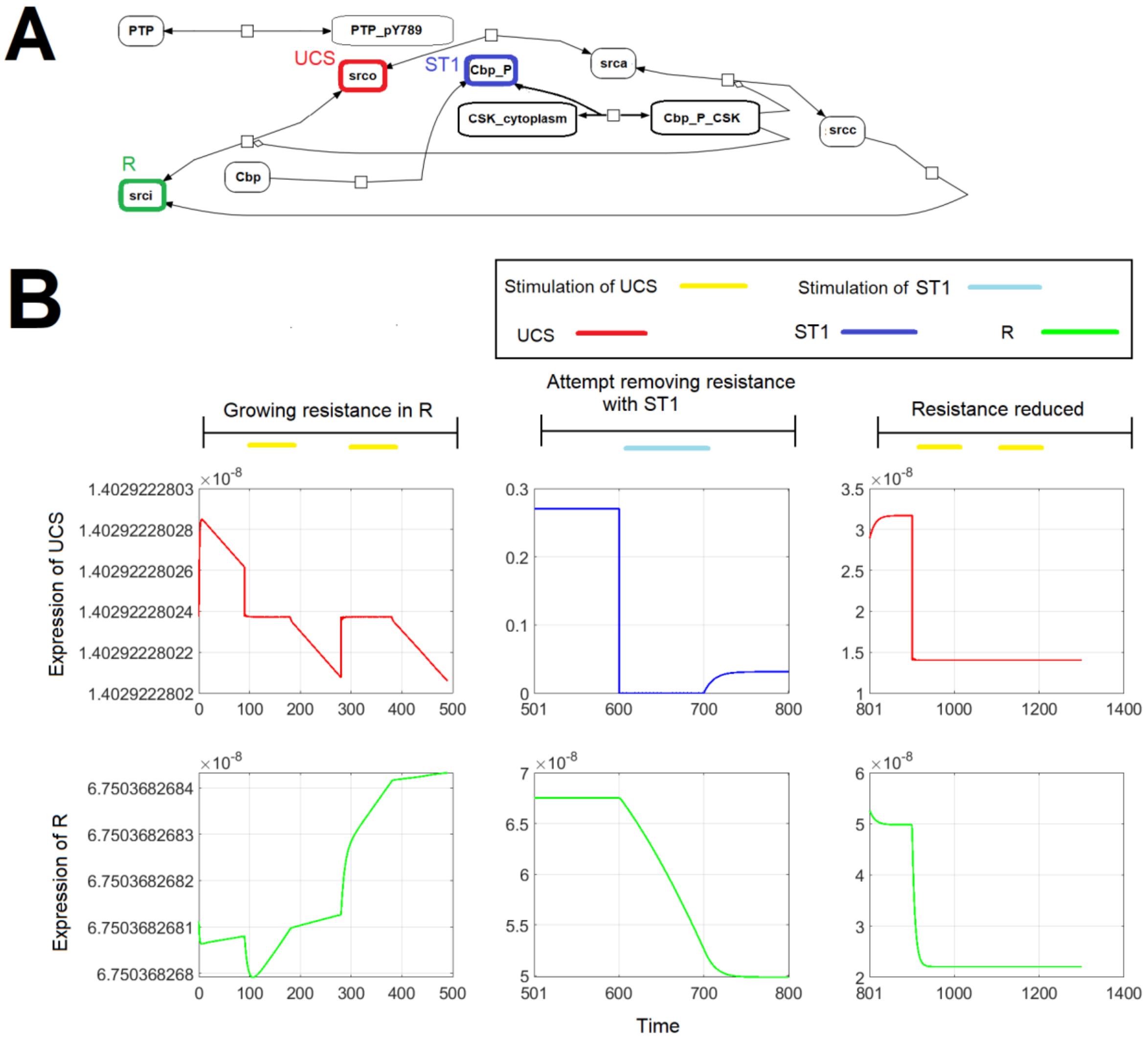

Figure S18. An example of sensitization in R, broken by stimulation of new stimuli. (A) The biological network taken from Biomodels website [5], comprise of a network having six nodes (proteins – Cyclin-P-CDC2, Cyclin-p-CDC2-P, Cyclin-P, Cyclin, CDC2-P and CDC2) their relations (edges), and the parameters. We mark the designated R (srci), UCS (srco), and ST1 (Cbp_P) with green, red, and blue, respectively. (B) Left, the last two stimulations of UCS and resulting sensitization of expression evolved in R. Here, down stimulation of UCS induced sensitization in R. Middle, the attempt breaking sensitization of R by stimulation of ST1 and resulting expression of R. Right, the two repetitive stimulations of UCS and resultant trajectory of R.

Software

Biomodels.zip – This file contains 3 functions per model for describing each of 35 models. The functions are a) executable .m file available from the Biomodels website, b) the function f() calculating derivative and c) initialize_ODE function for initializing species at the starting.

References

1. Edelstein, S.J., et al., *A kinetic mechanism for nicotinic acetylcholine receptors based on multiple allosteric transitions.* Biological cybernetics, 1996. **75**(5): p. 361-379.
2. Goldbeter, A., *A minimal cascade model for the mitotic oscillator involving cyclin and cdc2 kinase.* Proceedings of the National Academy of Sciences, 1991. **88**(20): p. 9107-9111.
3. Tyson, J.J., *Modeling the cell division cycle: cdc2 and cyclin interactions.* Proceedings of the National Academy of Sciences, 1991. **88**(16): p. 7328-7332.
4. Kholodenko, B.N., *Negative feedback and ultrasensitivity can bring about oscillations in the mitogen-activated protein kinase cascades.* European journal of biochemistry, 2000. **267**(6): p. 1583-1588.
5. Fuß, H., et al., *Bistable switching and excitable behaviour in the activation of Src at mitosis.* Bioinformatics, 2006. **22**(14): p. e158-e165.
6. Goldbeter, A., *A model for circadian oscillations in the Drosophila period protein (PER).* Proceedings of the Royal Society of London. Series B: Biological Sciences, 1995. **261**(1362): p. 319-324.
7. Hoefnagel, M.H., et al., *Metabolic engineering of lactic acid bacteria, the combined approach: kinetic modelling, metabolic control and experimental analysisThe GenBank accession number for the sequence reported in this paper is AY046926.* Microbiology, 2002. **148**(4): p. 1003-1013.
8. Ueda, H.R., M. Hagiwara, and H. Kitano, *Robust oscillations within the interlocked feedback model of Drosophila circadian rhythm.* Journal of theoretical biology, 2001. **210**(4): p. 401-406.
9. Leloup, J.-C. and A. Goldbeter, *Chaos and birhythmicity in a model for circadian oscillations of the PER and TIM proteins in Drosophila.* Journal of theoretical biology, 1999. **198**(3): p. 445-459.
10. Rohwer, J.M. and F.C. Botha, *Analysis of sucrose accumulation in the sugar cane culm on the basis of in vitro kinetic data.* Biochemical Journal, 2001. **358**(2): p. 437-445.
11. olde Scheper, T., et al., *A mathematical model for the intracellular circadian rhythm generator.* Journal of Neuroscience, 1999. **19**(1): p. 40-47.
12. Markevich, N.I., J.B. Hoek, and B.N. Kholodenko, *Signaling switches and bistability arising from multisite phosphorylation in protein kinase cascades.* The Journal of cell biology, 2004. **164**(3): p. 353-359.
13. Cao, Y. and J. Liang, *Optimal enumeration of state space of finitely buffered stochastic molecular networks and exact computation of steady state landscape probability.* BMC Systems Biology, 2008. **2**(1): p. 1-13.
14. De Caluwé, J., et al., *A compact model for the complex plant circadian clock.* Frontiers in plant science, 2016. **7**: p. 74.
15. Chickarmane, V., et al., *Transcriptional dynamics of the embryonic stem cell switch.* PLoS computational biology, 2006. **2**(9): p. e123.
16. Chickarmane, V. and C. Peterson, *A computational model for understanding stem cell, trophectoderm and endoderm lineage determination.* PLoS one, 2008. **3**(10): p. e3478.
17. François, P. and V. Hakim, *Core genetic module: the mixed feedback loop.* Physical Review E, 2005. **72**(3): p. 031908.

18. Muller, M., et al., *Network topology determines dynamics of the mammalian MAPK1, 2 signaling network: bifan motif regulation of C-Raf and B-Raf isoforms by FGFR and MC1R.* The FASEB Journal, 2008. **22**(5): p. 1393-1403.
19. Goldbeter, A., D. Gonze, and O. Pourquié, *Sharp developmental thresholds defined through bistability by antagonistic gradients of retinoic acid and FGF signaling.* Developmental dynamics: an official publication of the American Association of Anatomists, 2007. **236**(6): p. 1495-1508.
20. Ciliberto, A., et al., *A kinetic model of the cyclin E/Cdk2 developmental timer in Xenopus laevis embryos.* Biophysical chemistry, 2003. **104**(3): p. 573-589.
21. Cellière, G., et al., *The plasticity of TGF-β signaling.* BMC systems biology, 2011. **5**(1): p. 1-13.
22. Marhl, M., et al., *Complex calcium oscillations and the role of mitochondria and cytosolic proteins.* Biosystems, 2000. **57**(2): p. 75-86.
23. Martins, S.I. and M.A. Van Boekel, *Kinetic modelling of Amadori N-(1-deoxy-D-fructos-1-yl)-glycine degradation pathways. Part II—kinetic analysis.* Carbohydrate research, 2003. **338**(16): p. 1665-1678.
24. Vilar, J.M., et al., *Mechanisms of noise-resistance in genetic oscillators.* Proceedings of the National Academy of Sciences, 2002. **99**(9): p. 5988-5992.
25. Tyson, J.J., et al., *A simple model of circadian rhythms based on dimerization and proteolysis of PER and TIM.* Biophysical journal, 1999. **77**(5): p. 2411-2417.
26. Marwan, W., *Theory of time-resolved somatic complementation and its use to explore the sporulation control network in Physarum polycephalum.* Genetics, 2003. **164**(1): p. 105-115.
27. Rohwer, J.M., et al., *Understanding glucose transport by the bacterial phosphoenolpyruvate: glycose phosphotransferase system on the basis of kinetic measurements in vitro.* Journal of Biological Chemistry, 2000. **275**(45): p. 34909-34921.
28. Chelliah, V., C. Laibe, and N. Le Novère, *BioModels database: a repository of mathematical models of biological processes*, in *In Silico Systems Biology*. 2013, Springer. p. 189-199.
29. Le Novere, N., et al., *BioModels Database: a free, centralized database of curated, published, quantitative kinetic models of biochemical and cellular systems.* Nucleic acids research, 2006. **34**(suppl_1): p. D689-D691.
30. Li, C., et al., *BioModels Database: An enhanced, curated and annotated resource for published quantitative kinetic models.* BMC systems biology, 2010. **4**(1): p. 1-14.
31. Izquierdo, E., I. Harvey, and R.D. Beer, *Associative learning on a continuum in evolved dynamical neural networks.* Adaptive Behavior, 2008. **16**(6): p. 361-384.
